# Autonomous Waste Classification Using Multi-Agent Systems and Blockchain: A Low-Cost Intelligent Approach

**DOI:** 10.3390/s25144364

**Published:** 2025-07-12

**Authors:** Sergio García González, David Cruz García, Rubén Herrero Pérez, Arturo Álvarez Sanchez, Gabriel Villarrubia González

**Affiliations:** Expert Systems and Applications Laboratory, Faculty of Science, University of Salamanca, 37008 Salamanca, Spain; sergio.gg@usal.es (S.G.G.); david.cruz.garciaa@usal.es (D.C.G.); ruherrero@usal.es (R.H.P.); arturoas@usal.es (A.Á.S.)

**Keywords:** smart waste management, multi-agent systems, blockchain, intelligent classification

## Abstract

The increase in garbage generated in modern societies demands the implementation of a more sustainable model as well as new methods for efficient waste management. This article describes the development and implementation of a prototype of a smart bin that automatically sorts waste using a multi-agent system and blockchain integration. The proposed system has sensors that identify the type of waste (organic, plastic, paper, etc.) and uses collaborative intelligent agents to make instant sorting decisions. Blockchain has been implemented as a technology for the immutable and transparent control of waste registration, favoring traceability during the classification process, providing sustainability to the process, and making the audit of data in smart urban environments transparent. For the computer vision algorithm, three versions of YOLO (YOLOv8, YOLOv11, and YOLOv12) were used and evaluated with respect to their performance in automatic detection and classification of waste. The YOLOv12 version was selected due to its overall performance, which is superior to others with mAP@50 values of 86.2%, an overall accuracy of 84.6%, and an average F1 score of 80.1%. Latency was kept below 9 ms per image with YOLOv12, ensuring smooth and lag-free processing, even for utilitarian embedded systems. This allows for efficient deployment in near-real-time applications where speed and immediate response are crucial. These results confirm the viability of the system in both accuracy and computational efficiency. This work provides an innovative solution in the field of ambient intelligence, characterized by low equipment cost and high scalability, laying the foundations for the development of smart waste management infrastructures in sustainable cities.

## 1. Introduction

Urban solid waste management has become one of the most urgent environmental problems of the 21st century [1]. Every year, the world generates more than 2010 million tons of solid waste, and estimates indicate that this figure will increase by 70% by 2050 if effective measures are not adopted [2]. This growth is closely linked to population growth, urban development, and increasingly unsustainable consumption habits [3].

Despite efforts in selective collection, many municipalities have significant inefficiencies in the management of urban solid waste. Only a small number of municipalities achieve optimal levels of recycling, and higher unit costs are observed in those with a lower proportion of separate collection, suggesting limited waste use and poor economic efficiency in the public collection service [4]. Despite efforts in selective collection, many municipalities have significant inefficiencies in the management of urban solid waste, which contributes to the contamination of the soil around poorly managed landfills, as has been documented in several regions, where the lack of leachate control and inadequate waste disposal generate a considerable environmental impact [5]. Milan, Italy and Madrid, Spain, for example, are among the cities with the highest mortality rates due to air pollution, specifically waste incineration and urban traffic [6]. This situation highlights the urgent need to apply more efficient and sustainable technologies in waste management at the national level.

The accumulation of waste that is not managed correctly represents a serious ecological problem, as much of this waste ends up in open landfills or is burned, generating toxic emissions and greenhouse gases that contribute to climate change [7]. In addition, poorly managed waste directly affects public health by contaminating water, air, and soil sources, leading to the proliferation of diseases [8].

One of the biggest challenges in waste management is that, on many occasions, citizens do not properly separate waste at the place where it is generated. This first classification, i.e., depositing each type of waste in its corresponding container, is essential to ensure efficient recycling [9]. However, in practice, this task is often carried out incorrectly or omitted altogether. In addition, automatic classification systems that could complement or correct these errors remain scarce or technologically limited, even in urban environments in developed countries [10].

As a result, tons of recyclable materials such as plastics, glass, metals, or paper end up being wasted [11]. This means not only a loss of material resources, but also a significant economic loss, as management costs increase and circular economy opportunities are wasted [12]. In addition, this problem significantly limits the possibility of achieving the Sustainable Development Goals (SDGs) in terms of responsible consumption, climate change mitigation, and environmental health [13].

In this context, waste management represents a global challenge that requires new technological solutions [14]. The United Nations Industrial Development Organization (UNIDO) has stressed that a circular and sustainable economy will only be possible if innovative technologies capable of optimizing recycling processes and reducing the environmental impact of our activities are incorporated [15].

In recent years, technologies such as automation, the Internet of Things (IoT), and artificial intelligence (AI) have begun to transform multiple industry sectors. These tools, which allow large amounts of data to be analyzed and decisions to be made autonomously and very quickly, are also being applied to waste treatment with increasingly promising results.

In response to this need, solutions based on intelligent systems have emerged that use computer vision, specialized sensors, and machine learning algorithms to automatically identify and sort different types of waste. These approaches not only reduce human intervention but greatly increase the efficiency and accuracy of recycling, making it viable even in resource-limited settings.

This paper presents a practical solution to these problems in waste management by developing a low-cost smart sorting bin, designed with the aim of being accessible and functional even in computationally limited resource environments. The proposal combines technologies such as computer vision, inductive and capacitive sensors, and an artificial intelligence model trained with real data to identify, analyze, and classify different types of waste. Once recognized, the materials are automatically diverted to the appropriate container using a precision motorized system.

This entire system has been implemented within an environment based on a multi-agent system (MAS) [16], a distributed architecture that allows the recycling process to be divided into autonomous and intelligent parts that interact with each other to make collaborative decisions. In the architecture of this research work, each module of the system (detection, classification, decision, and mechanical action) acts as an agent with specific capabilities, which not only improves the overall efficiency of the system but also facilitates scalability, maintenance, and adaptation to new usage scenarios.

The fundamental purpose of this project is to bring technology closer to sustainability, promoting a culture of recycling based on data, automation, and efficiency. In addition, it aligns with several of the SDGs, including SDG 9 (Industry, Innovation and Infrastructure), SDG 11 (Sustainable Cities and Communities), SDG 12 (Responsible Consumption and Production), and SDG 13 (Climate Action). The initiative seeks to demonstrate that it is possible to develop affordable, reproducible, and real-impact technological solutions, even with limited resources.

This paper presents several relevant contributions to the field of smart waste management from a technological and scientific perspective. A distributed architecture based on multi-agent systems (MASs) is proposed for the automation of the waste sorting process, integrating technologies such as computer vision, specialized sensors, and intelligent control in a modular, scalable, and adaptable system. This approach allows progress in the design of decentralized and cooperative solutions for smart urban environments. Likewise, a comparative evaluation of different versions of object detection models based on YOLO is carried out, contributing to the knowledge about their applicability in real waste classification scenarios. Another outstanding contribution is the incorporation of a private blockchain infrastructure, which introduces an innovative approach to guarantee the traceability, integrity, and transparency of system events, with the potential to be replicated in other circular economy applications. In addition, as part of the work, a new specific dataset for the recognition of urban waste is proposed, opening a complementary line of research focused on large-scale environmental monitoring using artificial intelligence. Finally, future extensions of the system are proposed, including the incorporation of reinforcement learning techniques, new sensors for the classification of complex waste, and the creation of collaborative networks of smart bins, which reinforces the potential of this approach to evolve towards more autonomous, connectable, and sustainable solutions.

This article has been structured as follows: Section 1, Introduction, presents the global context of waste management. Section 2, State of the Art, reviews previous research and related projects, focusing on both smart waste sorting systems and their potential applications. Section 3, System Architecture, describes in detail the proposed system based on a multi-agent architecture, including the hardware components, internal modules, communication flow, and decision-making mechanism. Section 4, Case Study, analyzes the performance of the system through a practical evaluation of its classification capacity. Section 5, Results, presents the achievements obtained in the development of the prototype and the performance metrics of the artificial intelligence model. Section 6, Discussion, details the main challenges encountered during the development of the prototype, including the collection and quality of data from garbage collection, and their implications for the results. Section 7, Limitations, exposes the technical and contextual constraints of the proposed approach. Finally, Section 8, Conclusions and Future Lines, summarizes the main findings and proposes possible improvements, with a discussion of their potential impacts on urban waste management.

## 2. State of the Art

Machine vision, especially when combined with deep learning techniques, has demonstrated superior performance in the visual identification of waste [17]. Models trained with multiclass datasets such as DenseNet or ResNet [18] allow solid waste to be sorted with high accuracy, even in uncontrolled environments [19]. Projects based on EfficientNet [20] and others, such as ECCDN-Net [21], demonstrate how machine vision can be used to categorize household or industrial waste with promising results.

However, such approaches often require high computational resources or optimal lighting conditions, which limit them in low-cost or uncontrolled environments. Unlike these jobs, our system employs a YOLOv12 model optimized for limited hardware, maintaining adequate accuracy even in low-light situations or partially visible debris.

However, many of these applications are designed for industrial recycling infrastructures or municipal facilities, where robots, conveyor belts, and considerable computational capacity are available [22].

The system proposed in this article distances itself from these robust but expensive architectures by integrating computer vision into a low-cost physical platform, aimed at being used by anyone without the need for technical intervention, something little explored in the current literature.

In contrast to systems such as those presented by [20,21], which depend on specific infrastructure or technical support, our proposal seeks to democratize waste sorting through an autonomous, accessible, and replicable device, even in educational or community contexts [23].

In those cases where machine vision is not as accurate, for example, with dirty, deformed, or visually ambiguous debris, capacitive and inductive sensors have proven to be much more reliable. These sensors allow the presence of metals to be detected using electromagnetic fields, while capacitive ones identify materials by their dielectric constant [24,25]. These properties have been exploited in projects such as SmartBin or RFID systems in smart cubes [26,27].

Unlike these projects, where physical sensors act as support modules and rely on expensive technologies such as RFID, our system employs them as primary sources of information, eliminating the need for tagging and reducing operational costs. This allows for improved overall system robustness without sacrificing accessibility.

In contrast, the system proposed in this paper actively integrates inductive and capacitive sensors as primary sources of information, enabling reliable decisions without relying solely on images. This integration improves not only the accuracy but also the robustness of the system in varying environmental conditions, such as poor lighting or contaminated waste.

One of the least explored contributions in waste classification is the use of MASs. In contexts where multiple sensors, algorithms, and actuators must cooperate in a coordinated manner, they offer decisive advantages in terms of scalability, modularity, and resilience against partial failures [28,29].

While they have been successfully applied in urban logistics and collaborative robotics, their use in low-cost and home-based devices is virtually non-existent. Our approach proposes a decentralized architecture in which each module of the system acts as an agent, allowing local adaptation and reduction in failures without requiring complex infrastructure.

Our proposal includes a lightweight multi-agent system where each component of the sensor (system, camera, motor, and decision logic) acts as an agent with limited autonomy, allowing flexible and adaptive behavior without the need for constant monitoring or complex networks. This allows, for example, the system to self-regulate in the face of changing conditions or even learn through the feedback accumulated in databases, an aspect that is little addressed in current work.

Many of the systems reviewed, especially in academic settings, are focused on proofs of concept with low portability. For example, solutions with drones, robotic arms, or hyperspectral classifiers achieve high accuracy, but have high technical and economic requirements [30,31].

Faced with these sophisticated proposals, but not very viable outside the laboratory, our system seeks portability, scalability, and replicability. It thus provides a realistic and practical solution to be used in real scenarios with limited resources.

Other devices, such as smart cubes with weight sensors or RFID, are limited to recording information without making active decisions [32] to make recycling a simpler process.

In addition, another critical and often underestimated aspect is the traceability and security of the information generated from these classification processes [33]. The ability to robustly obtain, store, and analyze the detailed history of detected and classified items is not only critical for continuous system optimization but also opens up new avenues for statistical analysis and decision-making in waste management on a larger scale. Smart waste sorting systems generate a considerable volume of data, such as the type of material detected or the date. Our system proposes to make use of a private blockchain based on Ethereum to guarantee the traceability and security of classified garbage information [34]. Geth, or Go Ethereum, is the core technology of such a data structure, tasked with tasks such as blockchain initialization, node execution, network communication, and mining [35].

The proposed blockchain architecture uses Proof of Authority (PoA) as a consensus mechanism [36]. This mechanism consists of the use of authorized validators instead of computational tests or the ownership of cryptocurrencies. The cohesion and synchronization of the distributed network are achieved through the use of a bootnode. Its fundamental purpose is to act as an initial entry and discovery point for the rest of the nodes.

Each garbage classification, along with details such as date, time, and trash can identifier, can be recorded as an immutable transaction on the blockchain. This fundamental immutability feature ensures that once classification information is added and validated in a block, it cannot be altered or deleted. This results in a highly reliable and auditable data history, which is essential for accurate traceability of the waste stream, allowing exact tracking of what is sorted, when, and by what device. It also ensures integrity, safety, and efficiency in waste management.

Blockchain technology has emerged as a promising and disruptive solution for traceability, especially in supply chains, where trust, transparency, and security are critical. Although initially popularized in the financial field, its application has expanded into logistics and supply chain management thanks to its intrinsic characteristics: A Decentralized Distributed Ledger (DLT) allows consensus without a central authority, data immutability, real-time public accessibility, and time-stamped chronological recording [37]. These properties allow products to be traced holistically, creating a transparent and tamper-proof record from the source to the end user. By integrating with Internet of Things (IoT) devices, such as temperature sensors, RFID tags, GPS, and NFC, blockchain further enhances real-time monitoring and compliance with regulations such as the HACCP system, being especially useful in sectors such as food safety, agriculture, medicine, and industrial manufacturing [38].

There are already multiple practical applications in different sectors: traceability of food and agricultural products (grains, fish, and dairy), authentication of medicines, quality certification in the steel industry, and even product tracking as in the wood industry [39].

This technology allows each stage of the waste life cycle to be recorded in an immutable and decentralized way, reducing fraud and mismanagement [40]. Smart contracts automate processes such as payments and regulatory compliance, while tokenization incentivizes sustainable practices. Tokenization allows an asset to be converted into a digital asset (token), allowing it to be recorded and managed on a blockchain. In addition, it facilitates the monitoring of specific waste (plastics, electronics, textiles) and promotes a circular economy, maintaining the value of products and reducing waste generation. Innovations such as digital product passports and blockchain-based municipal management models are also explored [41,42]. Although challenges such as implementation costs and monitoring resistance remain, large-scale adoption can be viable and effective, especially in urban contexts with high volumes of waste.

At the algorithm level, a growing trend is supervised training of classifiers via CNN using specific residue imagery. DeepWaste and other mobile apps have shown that it is possible to identify waste with a simple photo taken from a smartphone [43,44].

However, in contrast to DeepWaste, which requires direct user interaction through a mobile app, our system fully automates detection, sorting, and recording, without manual intervention, which improves the user experience and promotes the passive adoption of sustainable practices.

The proposed system automates this process to the maximum, with a web application that allows the visualization of statistics but does not require additional interactions on the part of the user. In addition, the model can be continuously trained with new waste, generating an increasingly robust system from everyday use, without the need for manual reconfiguration or technical supervision.

Despite the important technical advances, most of the systems reviewed have recurrent limitations: an industrial or institutional approach that is not feasible for domestic use [45]; technical or unintuitive interfaces for the end user; the need for active intervention or manual labeling; and lack of integration between vision, sensors, and real-time decision-making [46].

Faced with these limitations, the system developed for this article represents an unprecedented hybrid solution in the literature: It combines computer vision, physical sensors, and MAS-based logic in a compact, accessible, and replicable physical device. The classification is carried out ubiquitously for the end user.

Our system, by comparison, excels at prioritizing accessibility, low cost, and ease of use, moving away from complex industrial models. Unlike rigid systems, we use lightweight multi-agent systems (MASs), giving each component autonomy and the ability to learn with use, a little-explored characteristic in domestic solutions. And to ensure trust, a private blockchain ensures the traceability and immutability of classification data, which is a crucial novelty in this type of application. In essence, our project proposes a hybrid, compact, and replicable device that fuses machine vision (YOLOv12) with physical sensors and MAS logic, all with the transparency of blockchain, seeking to make recycling passive and accessible to all.

While other research aims to increase the accuracy or broaden the range of waste, our project prioritizes usability, low cost, and practical applicability in educational, community, or domestic contexts. Thus, it is presented as a truly accessible solution that contributes to recycling in a passive way, eliminating barriers to entry for the common user and facilitating more widespread adoption.

## 3. System Architecture

This section is divided into different subsections. It will describe all the significant parts present within the system, including the multi-agent system, the physical components for implementation, the machine vision system for object detection, and the system implemented for traceability. All these components work in coordination through intelligent agents that collaborate in real time to offer an efficient solution.

### 3.1. Multi-Agent System

**Figure 1:**
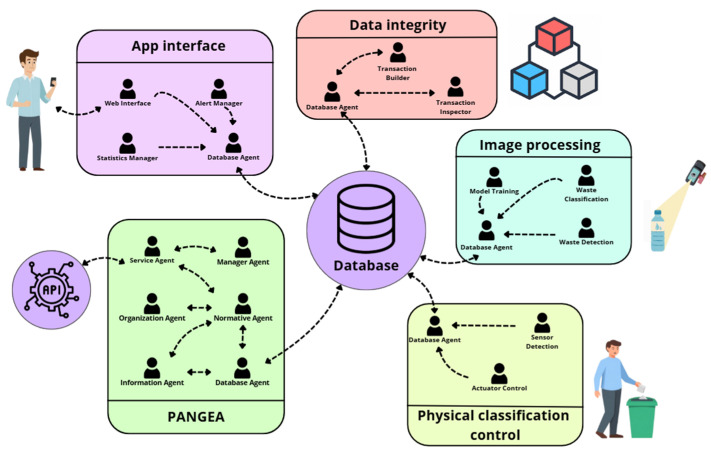
Multi-agent system of the proposed architecture.

As shown in Figure 1, the architecture of the designed system is composed of a series of virtual organizations making use of the PANGEA platform [47,48] and is designed to address the various tasks needed. The upper part of the figure groups the agents that make up the PANGEA platform, which acts as the management and control core of the multi-agent system.

PANGEA (Platform for Automatic coNstruction of orGanizations of intElligent Agents) is a multi-agent architecture specially designed to build virtual organizations (VOs). Unlike other platforms such as JADE, PANGEA is focused from its core on organized and hierarchical agent management, which makes it very suitable for complex and collaborative scenarios. The main advantages of using PANGEA for this job are as follows:

- Native support for virtual organizations: PANGEA not only manages individual agents, but also entire organizational structures. It allows us to model roles, norms, relationships, and collective objectives, facilitating the design of more natural and scalable systems.
- Use of RFC-based communication protocols: Unlike other environments that use specific protocols such as FIPA-ACL, PANGEA adopts an approach based on the principles of RFC (Request for Comment) protocols, similar to those used in web services. This improves interoperability, readability, and integration with external systems. In addition, it allows for greater efficiency on computationally limited devices.
- Modularity and functional decoupling: PANGEA components are highly modular, allowing them to be maintained and extended with ease. Each module can be upgraded or replaced without affecting the entire system.
- Support for complex organizational hierarchies: Organizational structures can be built with multiple levels (parent organization, sub-organizations, etc.), which is especially useful in contexts such as social simulation, dynamic distributed environments, or rescue systems.

The use of an MAS based on virtual organizations offers multiple benefits, including the following:

- Structured scalability: Instead of having a disorganized network of agents, VOs group agents according to roles and tasks, making it easier to manage the system at scale.
- Ease of coordination and delegation: Roles allow tasks to be assigned without relying on specific agents, simplifying task redistribution and improving adaptability.
- Organizational autonomy: Organizations can make internal decisions and adapt their behavior, promoting the autonomy and flexibility of the system.
- Management of common regulations and policies: Rules can be established at the organizational level that regulate the behavior of agents, ensuring consistency in their interactions.

PANGEA implements a communication system inspired by RFC1459 standards [49], such as those used in network protocols (e.g., HTTP). This offers several advantages:

- Interoperability: Standard-structured messages allow PANGEA to easily communicate with other external systems, including APIs and web services.
- Standardization: Standardization facilitates the understanding, traceability, and maintenance of messages between agents, as they follow widely known and documented formats.
- Simpler integration: Using a developer-friendly protocol makes it easier to integrate with other technologies without requiring learning more restrictive protocols like FIPA-ACL.

JADE (Java Agent Development Framework) is one of the most well-known and used platforms when it comes to building multi-agent systems. It is an open-source project written in Java that follows the specifications of FIPA (Foundation for Intelligent Physical Agents), especially the part that defines the language of conversation between agents: the FIPA-ACL. Thanks to this, JADE offers a set of tools that allow agents to create, implement, and communicate in a distributed network. Its design, however, focuses mainly on the operation of single agents and does not include more elaborate patterns that mimic a complete virtual organization as standard. While an experienced developer can build those hierarchies, you need to add extra modules and make adjustments to the configuration. Additionally, the dialect of communication it uses is still very robust, although it is less accessible to those who are used to the open APIs and JSON messages of current web technologies.

Table 1 compares JADE, which is the most widely used MAS, and PANGEA so that readers can easily understand their main differences.

The agents that make up the case study make use of virtual organizations using PANGEA. One of the virtues of this MAS is the supervision and discovery of services offered to the rest of the elements of architecture in a scalable and distributed way. The agents that make up the case study are the following:

- Service Agent: This agent exposes the system’s internal services to the outside through a REST API interface, allowing web, mobile, or third-party systems to interact with the multi-agent environment without needing to know its internal structure. It acts as a gateway for integration with external platforms.
- Manager Agent: The main function of this agent is continuous monitoring of the system. It carries out regular checks to detect loading problems, communication failures, or malfunctions of the rest of the agents. In the event of an error, you can activate recovery or task redeployment mechanisms to maintain system stability.
- Organization Agent: This agent is in charge of creating, configuring, and monitoring virtual organizations within the system. It supervises the correct functioning within the organization and ensures that each one complies with the established rules. In addition, it manages communications and facilitates the scalability of the system.
- Normative Agent: This agent ensures compliance within the multi-agent system. It defines and applies the rules of interaction, controlling who can communicate with whom, under what conditions, and at what times. It also restricts access to services when certain security conditions are not met.
- Database Agent: This is the only agent authorized to interact directly with the system database. It takes care of all read, write, and update operations, ensuring that the data is correct and consistent. Any other entity that wants to store or access the information must make its request through this agent.
- Information Agent: This agent keeps an up-to-date record of all active agents and the services they offer. When a new agent enters the system, it registers, and when an agent requires a service, it queries this agent to find out who provides it. It is key to finding and coordinating system services efficiently. It is what is commonly called the white pages agent, which is a kind of directory of services that lists the services offered by the architecture and the agents responsible for its functionality.

Next, the other virtual organizations that make up the platform are described, detailing their functions and how they contribute to the proper functioning of the whole.

- Physical sorting control is a virtual organization that brings together the agents responsible for interacting directly with the physical components of the system, such as sensors, actuators, and weighing mechanisms. Its main objective is to physically classify waste autonomously and safely.
  - Actuator Control: This agent is responsible for driving the mechanical components of the system, such as the motors that move the sorting box and the servo motors that open the discharge gates. It executes the orders issued by the decision agent and ensures the correct execution in each classification.
  - Sensor Detection: This agent coordinates the reading of data from physical sensors, such as the capacitive and inductive sensors, which allow the identification of the type of material (paper, plastic, and metal, among others).

▪ The image processing organization is responsible for the detection, analysis, and visual classification of waste. It uses advanced machine vision and machine learning techniques to extract relevant features from objects, allowing it to accurately identify and classify types of materials. This process is essential to validate the information obtained by other agents (the agents associated with the sensors), improving the efficiency and accuracy of the system in the management and classification of waste.
  - Model Training: This agent is responsible for training visual classification models using labeled datasets. It periodically updates the model to improve the accuracy of the system over time, adding new images of real waste.
  - Waste Detection: This agent is responsible for detecting the presence of residue in the image captured by the camera. Once the object is located, it extracts information about its shape, color, and texture, and delimits its contour to facilitate its classification.
  - Waste Classification: This agent uses a pre-trained model to classify waste into a specific category (plastic, glass, paper, organic, etc.). It combines visual information with sensory data and applies different techniques to make confident decisions, even in ambiguous situations.

▪ The organization in charge of data integrity aims to guarantee the traceability, transparency, and immutability of all the information generated throughout the classification process. To carry out this, it relies on blockchain technology, which allows each action and decision of the system to be recorded in a secure and verifiable way, avoiding manipulations and ensuring trust in the stored data.
  - Transaction Builder: This agent constructs the transactions that contain the sorting data: waste type, date, device (sorting machine), etc. This data is encapsulated in a format prepared for storage on the blockchain.
  - Transaction Inspector: This agent is responsible for validating that each transaction generated complies with the format and security regulations before being sent to the blockchain network. Only those that pass these checks are recorded, which protects the integrity of the system and prevents the addition of erroneous or manipulated information. In addition, if necessary, it also has the ability to retrieve recorded information, facilitating subsequent audits or verification processes.

▪ The organization is in charge of the user interface that centralizes all the mechanisms of interaction with the end user, as well as the presentation of the data generated by the system. Its main function is to offer accessible and efficient interfaces that allow both ordinary users and institutional managers to consult information and monitor the status of the system.
  - Web Interface: This agent represents the main graphical interface of the system, which is accessible by a browser. It allows you to start sorting, see the status of each drawer and its percentage of filling, and view graphs, among other things. It makes the system easy to use so that anyone can adopt it without difficulty.
  - Alert Manager: This agent monitors critical events (such as sorting errors or system overload) and generates automatic alerts in real time. These notifications can be sent to system administrators.
  - Statistics Manager: This agent is in charge of processing and presenting usage statistics, such as the number of wastes sorted or the percentage of filling of the containers. This information is extracted from the database and dynamically updated to provide visual and comparative metrics.

This structure, based on virtual organizations, allows each group of agents to act in a specialized but coordinated way, maximizing the system’s ability to adapt. In addition, the use of intelligent agents allows each component to learn or be replaced in isolation, without affecting the overall operation. This architecture, being designed to be replicable, accessible, and low-cost, is presented as a viable solution for this project.

#### Operation of the Multi-Agent System

At the physical and operational level, the multi-agent platform based on PANGEA is deployed on a distributed system composed of two levels: a microcomputer (Jetson Nano), where the logic of the multi-agent system and virtual organizations is executed, and a microcontroller (ESP8266) that interacts directly with the sensors and actuators of the classification environment. This separation between the cognitive layer (intelligent agents) and the physical layer (embedded devices) ensures a modular, scalable, and fault-tolerant architecture.

The microcomputer acts as the main reasoning node, coordinating the actions of the agents organized in the different VOs. These agents communicate through structured messages in JSON format, transmitted through efficient protocols such as WebSocket, HTTP, or MQTT, following the RFC1459 specification. This standardized communication model facilitates interoperability with other external systems and ensures seamless integration with web technologies.

From a functional point of view, the system operates following a distributed flow of decision-making. Waste sorting is conducted through a data fusion process, in which sensory readings (capacitive and inductive) and visual analysis based on machine learning are integrated. The images captured by the camera are processed locally using the YOLO model.

The sorting decision is validated by a control agent who combines the visual and physical results and issues an order to the Actuator Control agent, which activates the necessary mechanisms to deposit the waste in the corresponding container. Subsequently, a structured event is generated and sent to the data integrity VO. There, specialized agents (Transaction Builder and Transaction Inspector) encapsulate the information in a transaction that is recorded on a private blockchain network, ensuring the traceability and immutability of the process.

In parallel, the UI VO maintains a real-time display of the system status. Through the Web Interface agent, users can initiate classifications, view the status of compartments, receive alerts managed by the Alert Manager agent, and analyze statistics compiled by the Statistics Manager.

### 3.2. Physical Components of the Prototype

**Figure 2:**
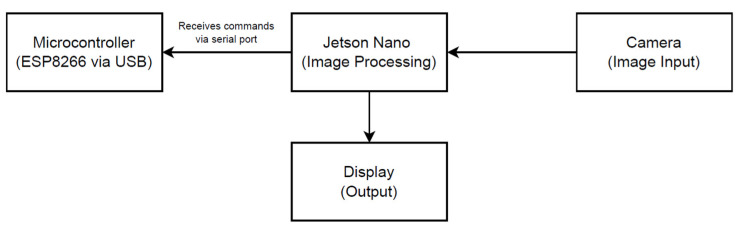
List of system components.

The prototype built for the realization of the case study consists of an automated low-cost bin designed to carry out, in a completely autonomous way, the physical classification of urban solid waste. The system integrates mechanical, electronic, sensorization, and communication components in a closed-loop architecture, aimed at operating without the need for direct human intervention and with operational feedback capacity. Figure 2 shows a diagram of how the components described below are related.

From a structural and functional point of view, the system is composed of a set of coordinated elements that allow an efficient flow in the waste sorting process. Initially, two DC motors control the opening of the safety gate and the initial displacement of the drawer that transports the waste. This movement is carried out on a timing belt, driven by a NEMA 17 stepper motor, whose speed and direction are managed by an L298N controller module.

Once the waste is introduced and the drawer reaches the analysis area, the sensorization subsystem is activated, consisting of a high-definition camera and specialized physical sensors. The camera used is a Logitech Brio, chosen for its ability to capture images in 4K Ultra HD resolution (up to 4096 × 2160 pixels), with autofocus and high dynamic range (HDR), which are essential features to ensure the sharpness and precision necessary in classification tasks using computer vision. This camera allows you to identify fine details such as texture, color, shape, and edges, which are key aspects to distinguish between visually similar objects.

The processing of the captured images and the classification model is carried out locally on a Jetson Nano microcomputer, which acts as the computational core of the system. In addition, this device manages information from the sensors, which is transmitted by an ESP8266 microcontroller. Finally, the Jetson Nano, through the agents, is also responsible for sending the information to the microcontroller to generate the movement in the motors and deposit the waste based on the information received by the sensors and the predicted data.

The sensors used are a capacitive sensor that determines the dielectric constant of the material, helping to discriminate non-metallic elements such as plastics and paper, and an inductive sensor that identifies the presence of metals through variations in electromagnetic fields. This data is integrated by the multi-agent system to validate or correct the visual classification.

The system is centralized by an ESP8266 microcontroller, which manages the communications between all the modules, the execution of the logic cycle, and the sending of data to the Jetson Nano, which is then in charge of sending that data to the cloud. From this interface, the capacity of each container of each of the devices that are installed can be consulted in real time.

**Figure 3:**
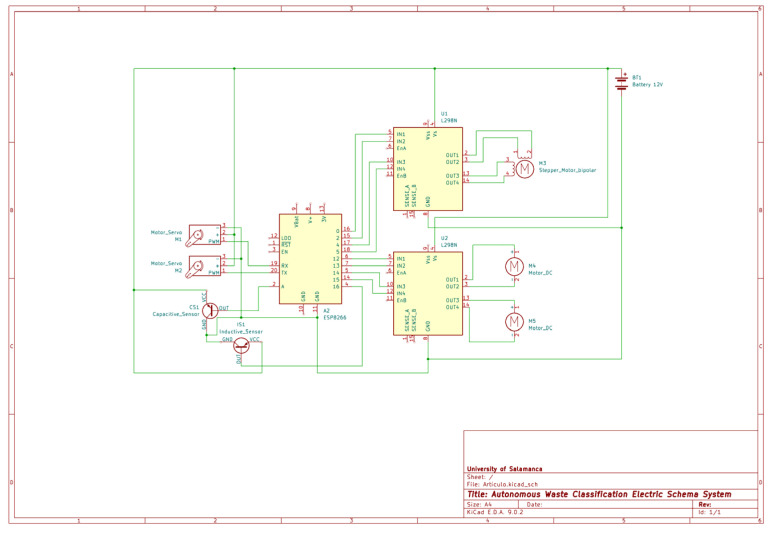
Electrical diagram of the system.

This design represents an accessible, replicable, and scalable solution that is suitable for implementation in domestic, educational, or institutional spaces that seek to automate the recycling process effectively, passively, and without technical barriers for the end user. Figure 3 shows the electrical diagram and the interconnection of the components with the microcontroller.

Below, a breakdown of the components used in the system is presented along with their approximate price in Table 2, in order to demonstrate its economic viability and that it is a low-cost solution compared to other alternatives on the market.

**Figure 4:**
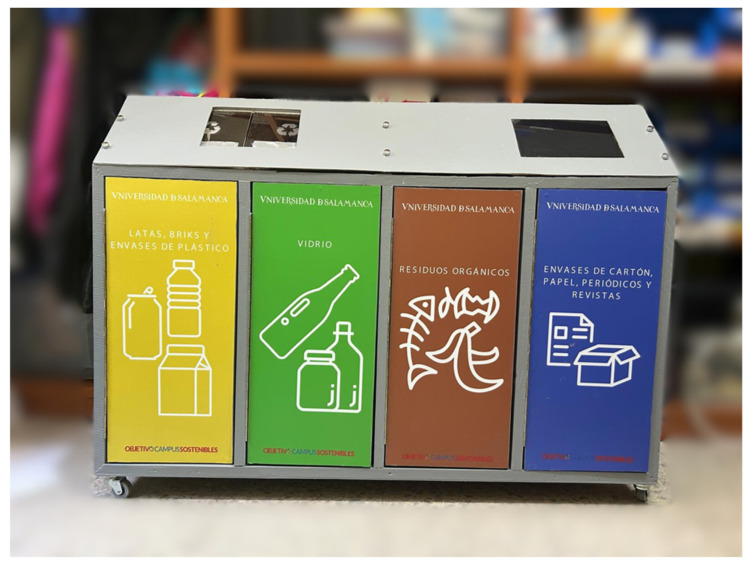
Final machine prototype.

In order for the reader to better understand the operation of the mechanical part, a video of the operation of the prototype built can be seen at the link provided in [50]. In addition, an image of the finished prototype can be seen in Figure 4.

### 3.3. Intelligent Detection System

With the aim of automating waste sorting and reducing human intervention in the separation process, an intelligent detection system based on computer vision techniques was developed. In particular, the YOLO (You Only Look Once) architecture was used, which is widely recognized for its ability to perform real-time object detection with high accuracy.

During the model training and validation process, experiments were conducted on several versions of this architecture: YOLOv8, YOLOv11, and YOLOv12. Each of these releases incorporates successive improvements in inference speed, generalizability, and performance in varying lighting conditions and angles. These versions were evaluated on different subsets of labeled data, which allowed the selection of the most robust and efficient model for the proposed use case.

To train the model, a set of data was generated from images collected from various internet sources. This dataset, publicly available on GitHub [51] commit 678a007, consists of 3134 images that are manually labeled according to five types of waste: metal, plastic, paper, glass, and organic, reaching a total of 4872 annotations that are distributed as can be seen in Table 3.

The dataset was divided into three subsets: 75% for training, 11% for validation, and 14% for testing, which allows a robust evaluation of the model’s performance in different phases of learning. In addition, all images were subjected to a preprocessing process that included two threads:

- Auto-orientation of the images to ensure consistent alignment.
- Forced resizing to a resolution of 640 × 640 pixels by stretching, ensuring homogeneity in input dimensions without cropping relevant content.

During the validation phase, three versions of the model were trained and evaluated: YOLOv8, YOLOv11, and YOLOv12, each with progressive improvements in terms of computational efficiency, generalization, and performance under variable environmental conditions (lighting, viewing angle, background, etc.).

**Figure 5:**
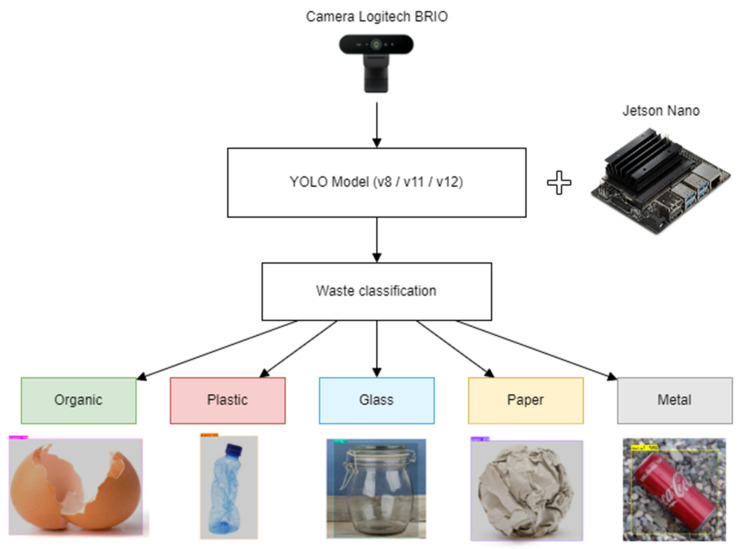
Operation flow of the intelligent detection system.

During operation, the system follows an automated flow as seen in Figure 5. A Logitech Brio camera captures in real time the image of the deposited waste; this image is processed by a YOLO model embedded in a local processing unit (Jetson Nano), which identifies the type of waste and assigns it a class. This sorting can be used to direct the waste to the appropriate compartment, record usage statistics, or even record the sorting event on a blockchain network to ensure traceability.

### 3.4. Traceability System

This case study addresses the design, development, and implementation of a waste traceability system using distributed ledger technology (blockchain) in conjunction with smart contracts deployed on the Ethereum network. The system’s architecture has been conceived with an approach aimed at transparency, immutability, and auditability of the data generated during waste management, from its point of generation to its final disposal.

Geth (Go Ethereum), a complete Ethereum client developed in the Go language, which allows the execution of nodes in a decentralized network and interaction with smart contracts, has been used for the operation of the blockchain network.

The core of a private blockchain network lies in creating nodes, through the creation of the Genesis block, establishing unique identities, configuring network parameters (such as consensus and resource allocation), and running the blockchain protocol through the Geth client. The Genesis block is a fundamental block for any blockchain, being the first block in the chain. It is imperative to create this block for the construction of a private network; otherwise, the general network of Ethereum is accessed. Each node validates transactions, stores a copy of the blockchain, and participates in consensus, contributing to a distributed, resilient, and secure environment.

The configuration of communication, usually facilitated by a bootnode, allows nodes to discover and connect to each other, forming a functional network. Each of the nodes on the blockchain in our system represents physical servers or computers configured to deploy the Ethereum software through Geth and maintain a complete and synchronized copy of the entire blockchain. This means that the information from all previous transactions and states is replicated and distributed among the network’s authorized participants, ensuring its persistence and resilience to failures. In order for transactions that record data on the blockchain to be valid and immutable, the network relies on a specific subset of these nodes, known as validator or miner nodes.

These nodes are responsible for validating transactions and generating gas, an essential element in any Ethereum blockchain to create blocks and insert them into the chain. When a new node joins the private network or an existing node is rebooted, it connects to the bootnode first. This element has a list of active nodes and their network addresses, making it easier for the newly connected node to discover and establish connections with other peers in the network.

It unifies the network itself, allowing each distributed copy of the blockchain on each node to efficiently synchronize with the others, ensuring that all participants have the same version of the data history. Thus, the information collected in each local instance of the blockchain is kept consistent and updated throughout the network through peer-to-peer communication facilitated by this initial discovery mechanism.

Readings of the data collected by sensors and the camera are encapsulated as structured events and recorded on a blockchain network through smart contracts to strengthen the validity and traceability of the system. This technological integration not only guarantees the accuracy and consistency of the information collected at each classification point but also adds a layer of security, immutability, and traceability to the entire process. Thus, the operational data used to calculate key metrics (such as daily and type classification rates or average hourly frequency) is supported by a decentralized infrastructure that allows transparent audits, interoperability with other systems, and an objective evaluation of the system’s performance in different contexts. Each logged event is encapsulated as a transaction that is sent to the smart contract. The latter contains functions that validate and store the data of each batch of waste, guaranteeing the integrity and consistency of the information through programmatic logic. Each record contains several fields that describe it, which are shown in Table 4.

Smart contract execution is designed to automatically reject any transaction that does not meet the validity requirements of the format, range, or logical consistency of the data. Once validated, the event is immutably stored on the blockchain, where each node maintains an up-to-date replica of the blockchain network. The smart contracts have been programmed in Solidity, a high-level language designed specifically for the creation of self-executing contracts on the Ethereum Virtual Machine (EVM).

To insert data into the Ethereum private blockchain, the process begins by setting up Geth. A Geth node is initialized with a custom Genesis file, which sets the rules and initial state of our local Ethereum network, allowing block mining and transactions. The logic for storing data resides in a Solidity smart contract. This contract is written by defining how the data will be saved (e.g., in a mapping or in a structure) and the functions to interact with it. Once codified, the contract is compiled. Finally, data insertion is carried out by invoking the smart contract functions from the graphical interface.

**Figure 6:**
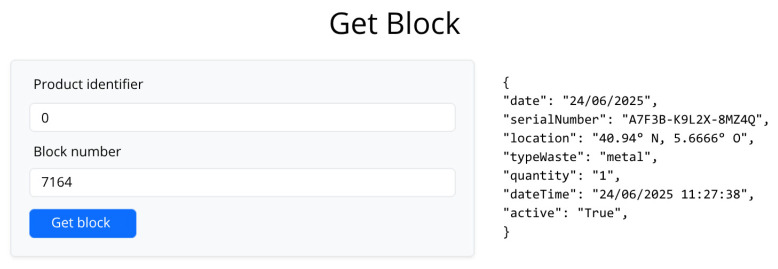
Example of residue visualization on the blockchain.

Each call to a function that modifies the state of the blockchain is packaged as a transaction. This transaction is signed by an account on our private network and sent to Geth. Once Geth processes it and a miner includes it in a block, the data is recorded in an immutable and decentralized way on the blockchain. The graphical interface allows us to visualize the entire process and the stored data. Figure 6 shows an example of visualizing the data of a piece of waste stored on the blockchain, with all the fields and related information.

### 3.5. YOLOv12

The model used that has offered the best results in the prediction is described below, which can be seen in Section 5 of the results. This version is the most recent of the well-known “You Only Look Once” family of models [52]. This model is not simply an incremental improvement, but an evolution that integrates advanced architectural principles to address the growing demands of machine vision applications. YOLOv12 distinguishes itself by adopting an attention-focused design philosophy, which is an improvement over previous versions that relied more on convolutional operations. This integration of attention mechanisms allows the model to not only capture high-resolution spatial features but also contextual ones, intelligently focusing on the most pertinent regions of the image.

**Figure 7:**
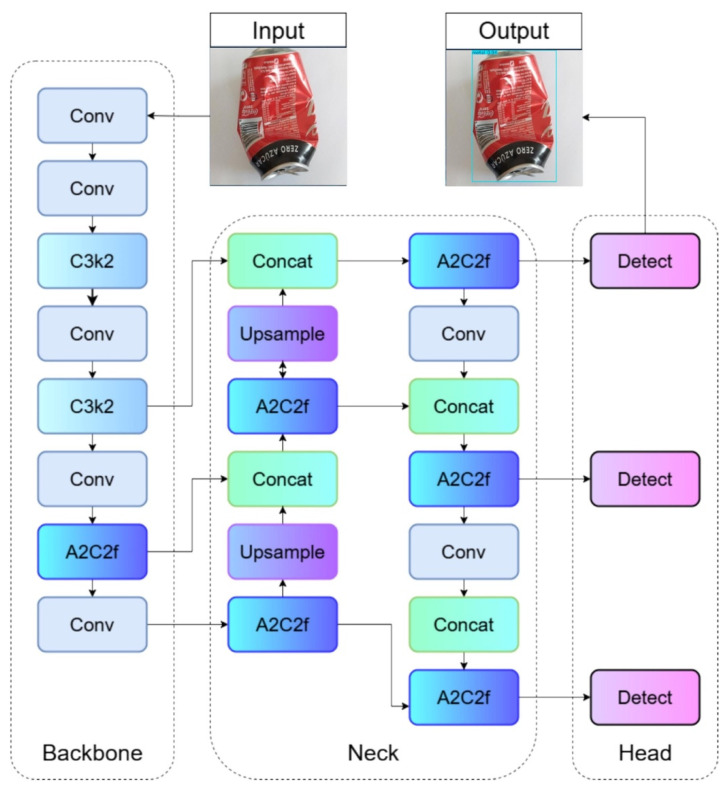
YOLOv12 model architecture.

The architecture of YOLOv12, shown in Figure 7, like that of its predecessors, is fundamentally organized into three interconnected components: the backbone, the neck, and the head [52]. However, YOLOv12 introduces significant refinements in each of these parts to optimize performance and efficiency, with a strong emphasis on the integration of advanced attention mechanisms.

The model’s backbone is initially responsible for extracting features from the input image. Its function is to process the raw image and produce feature maps at various scales, capturing everything from fine details to more abstract contextual information. In YOLOv12, the backbone was optimized with Residual Efficient Layer Aggregation Network (R-ELAN) blocks. These R-ELAN networks are designed to merge deeper convolutional layers with strategically placed residual connections, improving feature reuse and preventing gradient bottlenecks. This innovation allows the model to capture complex details of objects in different sizes and shapes, improving the robustness of feature extraction.

The neck adds and refines the multiscale features coming from the backbone before passing them to the sensing head. One of the most outstanding innovations in the YOLOv12 neck is the incorporation of an Area Attention mechanism. This module divides feature maps into segments, allowing the model to focus more efficiently on the critical regions of complex scenes. It is crucial to highlight that this area’s attention is accelerated by the integration of Flash Attention, a technique that minimizes memory access overhead, significantly reducing computational demand and closing the speed gap between attention mechanisms and traditional convolutional operations.

Finally, the head is the part of the model in charge of generating the final predictions. This is where the refined features are interpreted to produce the bounding boxes of the detected objects, their classes, and the associated confidence scores. YOLOv12 optimizes the loss functions to ensure high-precision object location and sorting, while maintaining the ability to operate in real time. This ensures that, despite the added complexities in the backbone and neck, the final output is fast and accurate.

## 4. Case Study

### 4.1. Experimental Design

In order to validate the effectiveness of the proposed system in real contexts, an empirical case study was designed to evaluate both the technical behavior of the prototype and its acceptance in diverse urban environments. The methodology was based on the installation of the prototype in four locations with differentiated socio-spatial characteristics, which were selected to represent different levels of population density, type of activity (educational, community, institutional, and public), and accessibility.

Each implementation remained operational for a period of one month, thus completing a total of four months of observation. This approach allowed a significant volume of data to be collected under real and heterogeneous conditions of use, ensuring greater generalizability of the results.

### 4.2. Data Collection and Metrics

Each prototype has been located in controlled environments such as educational centers, public spaces, institutions, and neighborhood communities. During the time of operation, the bin operated completely autonomously, recording and sorting waste in real time. Once a week, the compartments were physically emptied, at which time the operating data recorded by the ESP8266 microcontroller were also extracted.

The system has a category counting mechanism, which increments a specific counter each time a piece of waste is classified into one of the four categories configured: plastic, cardboard, glass, and organic. In addition to the cumulative count, the exact date and time of each qualifying event are automatically recorded. This data structure allows temporal analyses to be carried out on both a daily and weekly scale, in addition to establishing comparisons between locations.

Based on the data collected, the following four key metrics were defined to evaluate the behavior of use of the system and its impact on recycling practices in the different implementation contexts:

- Daily Sorting Rate by Waste at Each Location (DCRWL): This metric represents the daily amount of waste sorted, disaggregated by type of material and disposal point.
- Total Daily Sorting Rate by Location (TDCRL): This metric indicates the total number of pieces of waste sorted per day at each disposal point, without distinguishing types of material.
- Average Hourly Frequency by Location (HDIRL): This metric represents the average time at which sorting events occur by waste type. It allows the identification of behavioral patterns that serve to optimize the operation of the system (such as maintenance or emptying).
- Automatic sorting error rate: This metric assesses the accuracy and reliability of the system by identifying the proportion of waste sorted incorrectly.

These metrics provide a solid basis for conducting rigorous quantitative analysis of system usage in real-world environments. The detailed explanation of these indicators, together with their graphical visualizations and the analysis of their relevance, is presented in Section 6, Discussion, where their impact on the recycling rate is evaluated, differences between locations are identified, and correlations are established between the type of waste, frequency of use, and context.

Additionally, thanks to the use of a blockchain-based infrastructure, recorded events are traced in a secure and verifiable manner, allowing interoperability with analysis platforms, environmental incentive systems (based on tokens), and regulatory reporting frameworks.

### 4.3. Dataset and Training of the Classification Model

The automatic waste sorting system is based on a computer vision model trained on a specific dataset, adapted and labeled for the categories of plastic, cardboard, glass, and organic. The images were selected under criteria of visual representativeness and environmental variability, in order to maximize the robustness of the model in real environments.

For performance benchmarking, four versions of the YOLO algorithm were trained and validated: YOLOv8, YOLOv11, and YOLOv12. The training was carried out using data augmentation techniques and a 75/11/14 percentage partition of the whole (training/validation/test). Table 5 shows the total number of images in each set.

The models were evaluated using standard metrics in object classification and detection tasks, including the following:

- Precision: The ratio of correct predictions among all positive predictions.
- Recall: The ratio of correct predictions among all the actual positive elements.
- F1 score: Harmonic means between accuracy and completeness.
- mAP (mean Average Precision): A common metric in object detection that summarizes accuracy at different confidence thresholds.
- FPS: Metrics that measure the frames per second of the system, helping to calculate the inference time of the image.
- Confusion matrix: A visual representation of performance by category, which is useful for identifying common biases or errors.

The results obtained are analyzed in the next section, allowing the performance of each version to be compared and justifying the choice of the final model implemented in the prototype.

### 4.4. Setting up the Test Environment

The machine learning models developed were trained and evaluated in a local computing environment without the need for cloud services. The specifications of the equipment and software used can be seen in Table 6.

This hardware was suitable both for the training process of the YOLOv8, YOLOv11, and YOLOv12 models and for the execution of inferences in a reasonable time, allowing experimentation with different configurations and datasets efficiently.

### 4.5. Training the Model

To ensure an optimal configuration of the model and improve its performance, a grid search methodology was implemented for the selection of the most relevant hyperparameters: number of epochs, batch size, and learning rate. This strategy allows you to evaluate multiple combinations of values and select those that maximize the model’s performance metrics.

Specifically, the following hyperparameter combinations were tested:

- Epochs: 50, 100, and 200
- Batch size: 8, 16 (limited by RTX 4050 6 GB GPU memory)
- Learning rate: 0.1, 0.01, and 0.001

Table 7 below shows the best hyperparameters selected for each variant of YOLO. For a more detailed description of the full results and analysis, please refer to Section 5 (Results). In addition, Table 8 shows the parameters selected to be used in all the proposed YOLO variants.

### 4.6. System Traceability

The integration of blockchain technology is a fundamental architectural component that elevates the system beyond a simple sorting machine. The inclusion of blockchain technology is justified by the need to establish a recycling ecosystem that operates with unwavering trust, full transparency, and an automated and fair incentive system, addressing shortcomings inherent in traditional waste management models.

The blockchain plays a crucial role in providing immutable traceability of every recycling interaction. Every time a user deposits correctly sorted waste in the smart bin, this action is recorded as a verifiable transaction on the blockchain network. This transaction not only details the type of material (e.g., PET, aluminum, or glass) and its quantity (weight or volume), but also associates the action with a user identifier and a precise timestamp and location. This unalterable chain of records creates a complete and auditable history of recycled materials from their origin to the processing phases, eliminating any ambiguity or possibility of manipulation in the recycling data. This capability is vital to ensure that all stakeholders, from citizens to waste managers to recycling companies, can verify the authenticity and flow of materials.

Beyond traceability, the distributed and transparent nature of blockchain fosters radical transparency throughout the recycling cycle. Data on the volume of waste processed, the operational efficiency of the bins, and the patterns of user participation become accessible for a public and decentralized audit. This builds essential trust in sustainability reporting and allows local authorities to more accurately monitor compliance with recycling targets, while citizens can directly see the impact of their actions.

Perhaps the most transformative benefit to the user experience is the enablement of a tokenized incentive system. Thanks to smart contracts programmed into the blockchain, every successful deposit in the smart bin can trigger the automatic issuance of digital tokens to the user’s wallet. These tokens are not just points, but digital assets that can possess value, be exchanged for goods or services, or even represent credits in circular economy programs. This mechanism not only gamifies the act of recycling but also provides a direct, transparent, and automated stimulus to encourage sustainable behaviors, overcoming the limitations of manual and opaque incentive programs. In addition, the ability to record the quality and purity of deposited material directly on the blockchain allows incentives to be differential, thus fairly rewarding users who contribute better-sorted waste. In essence, the blockchain transforms the smart bin into a node of a network of trust that verifies, incentivizes, and makes transparent the entire recycling effort.

## 5. Results

In order to quantitatively evaluate the performance of the proposed system, this section presents the results obtained from the data collected during the case study. The metrics described in the previous section were applied to analyze the behavior of the system during use, as well as its capacity to detect and classify waste in real conditions.

In this section, the results of the classification model trained with different versions of YOLO (v8, v11, and v12) are presented, analyzing its accuracy, recall, F1 score, and mAP. The evaluation of these models allows us to determine the most suitable version for implementation in real environments, considering both accuracy and computational performance.

The results obtained are discussed based on their potential impact on recycling practices, the adaptability of the system to diverse contexts, and the effectiveness of the technologies involved (AI, multi-agent systems, and blockchain).

### 5.1. Results of Model Training

#### 5.1.1. Evaluation Metrics

The rigorous evaluation of the detection models trained in this work is based on a set of standardized metrics in the field of computer vision. These metrics allow you to accurately quantify the behavior of the model in the face of unseen images, as well as evaluate its ability to generalize, minimize errors, and offer robust predictions in different contexts.

One of the fundamental metrics is accuracy (1), which indicates the percentage of true positives among all the positive predictions made by the model. This metric is especially useful for assessing the reliability of detections: high accuracy suggests that the model makes few mistakes when labeling residuals that do not actually belong to the predicted class.

(1) $$Precision=\frac{TP}{TP+FP}$$

In parallel, completeness or recall (2) is considered, which measures the proportion of true positives over the total number of real objects of a class that appear in the images. In other words, this metric reflects how complete the model’s detection is: a high value implies that few residues were ignored or not detected.

(2) $$Recall=\frac{TP}{TP+FN}$$

The F1 score (3) provides a harmonic mean between accuracy and recall, which allows a balanced measure to be obtained in contexts where there is class imbalance or when it is equally important to avoid both false positives and false negatives. This metric is particularly relevant in tasks such as waste sorting, where certain errors can have practical consequences on the effectiveness of recycling.

(3) $$F1=2\cdot \frac{Precision\cdot Recall}{Precision+Recal}$$

On the other hand, the mAP (mean Average Precision) metric, widely used in detection models, evaluates the overall quality of the model by considering not only whether it detects correctly, but also how accurate its spatial prediction is with respect to the real object. Two variants have been taken into account in this study: mAP@50 (4) (where a 50% overlap between prediction and label is accepted) and mAP@50:95 (5) (averaging over multiple overlap thresholds), which allows performance to be measured in both lax and strict terms.

(4) $$AP=\int_{0}^{1}P\left(r\right)dr$$

(5) $$mAP=\frac{1}{N}\sum_{i=i}^{N}{AP}_{i}$$

Inference time per image, expressed in frames per second (FPS) (6), is composed of three main stages: (a) image preprocessing time (which includes operations such as scaling and normalization), (b) model forward propagation time, and (c) post-processing time (which encompasses tasks such as decoding predictions and non-maximum suppression).

(6) $$FPS=\frac{1000}{\left(a+b+c\right)}$$

Finally, the confusion matrices corresponding to each model are analyzed. These matrices allow us to visualize, for each class, how many items were correctly classified and where the errors were concentrated, revealing systematic patterns of confusion between categories such as paper and organic or plastic and glass.

#### 5.1.2. YOLOv8 Model Results

The YOLOv8 model was the first model evaluated in the framework of this study for the automatic classification of waste using computer vision. The model was trained for 50 epochs with a batch size of 8 and a learning rate of 0.001 (b8_lr0.001). For the evaluation, parameters were configured with a confidence threshold of 80%, a maximum overlap threshold (max. overlap threshold) of 30%, and an IoU threshold of 45%. These values ensured an appropriate balance between accurate detection and reduction in redundant predictions, maximizing the usefulness of each inference in a realistic environment.

In terms of overall performance, YOLOv8 achieved a mAP@50 (mean Average Precision at a 50% threshold) value of 82.9%, reflecting a solid ability to correctly locate and classify objects within the test set images. This metric summarizes the average accuracy obtained in each class by considering as positive those detections whose intersection with the actual annotation exceeds 50%.

In addition, an overall accuracy of 77.8% was recorded, indicating that the vast majority of the predictions made by the model were correct. This reflects a low proportion of false positives, i.e., cases where an object was misidentified or misclassified. Likewise, the recall or completeness reached 79.6%, which implies that the model was able to identify most of the residues actually present in the images, although with a small proportion of false negatives that must be taken into account.

**Figure 8:**
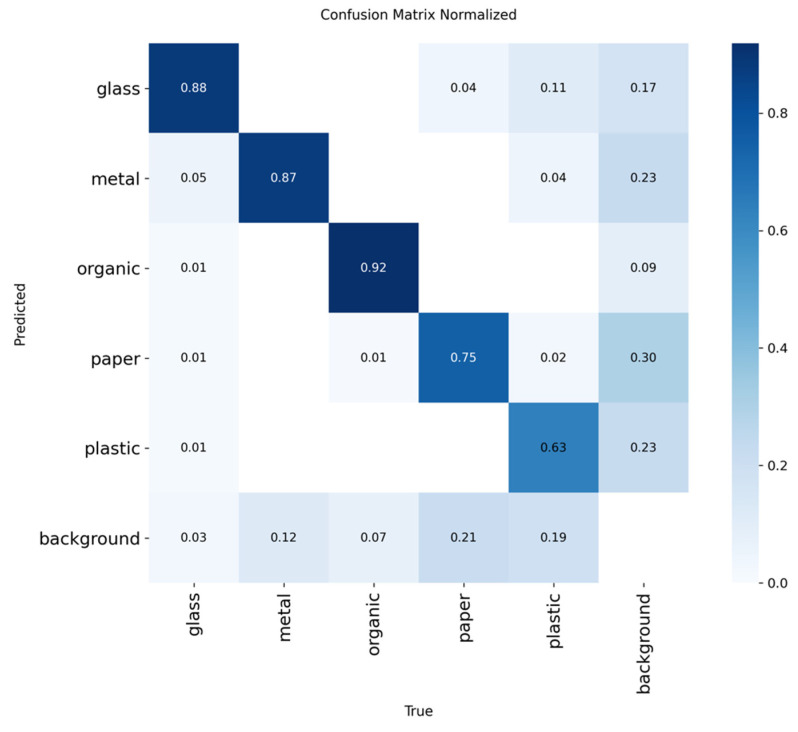
Confusion matrix generated for the YOLOv8 model.

To evaluate the performance of the YOLOv8 model in the residue classification task, an inference was carried out using the test dataset exclusively, as shown in Table 5. From the results obtained, a confusion matrix was obtained, which is shown in Figure 8, which allowed the precise identification of the true positive (TP) classifications, false positives (FP), false negatives (FN), and the total of real samples by class (GT).

This matrix allows us to clearly observe the relationships of successes and errors between the different types of residuals, facilitating the identification of patterns of confusion, especially between categories with similar visual characteristics.

With this information, the key evaluation metrics for each waste category were calculated: accuracy, recall, F1 score, mAP@50, and mAP@50:95. Table 9 summarizes these indicators, providing a detailed view of the model’s behavior by class, which is essential to understand its strengths and limitations in real application contexts.

The results show a good overall performance of the model in detecting different types of garbage. The organic and metal classes have the highest metrics, with accuracy and recall close to 90%, indicating a very reliable and balanced detection for these materials. Glass also has a solid performance, especially in recall (86.84%), suggesting that the model identifies most objects in this class, albeit with somewhat lower accuracy. On the other hand, paper and plastic have lower metrics, especially in recall and F1, which implies that the model has a harder time correctly identifying all objects in these categories. Notably, the mAP@50:95 for paper is the lowest (47.13%), indicating that performance at different IoU thresholds is less stable. These results suggest that, although the model is effective for most classes, it could benefit from further improvement and focused training in the paper and plastic categories to increase its accuracy and coverage.

#### 5.1.3. Results of the YOLOv11 Model

The YOLOv11 model was evaluated in the same way as the previous one, using the test suite of demanding evaluation parameters, namely a confidence threshold of 80%, a maximum allowable overlap of 30%, and an intersection threshold on junction (IoU) of 45%. In this case, the model was trained during 50 epochs with a batch size of 8 and a learning rate of 0.01; the hyperparameters were different from those used in YOLOv8. Under these conditions, YOLOv11 achieved a mAP@50 of 83.3%, reflecting significantly higher performance in detecting objects with acceptable spatial location.

**Figure 9:**
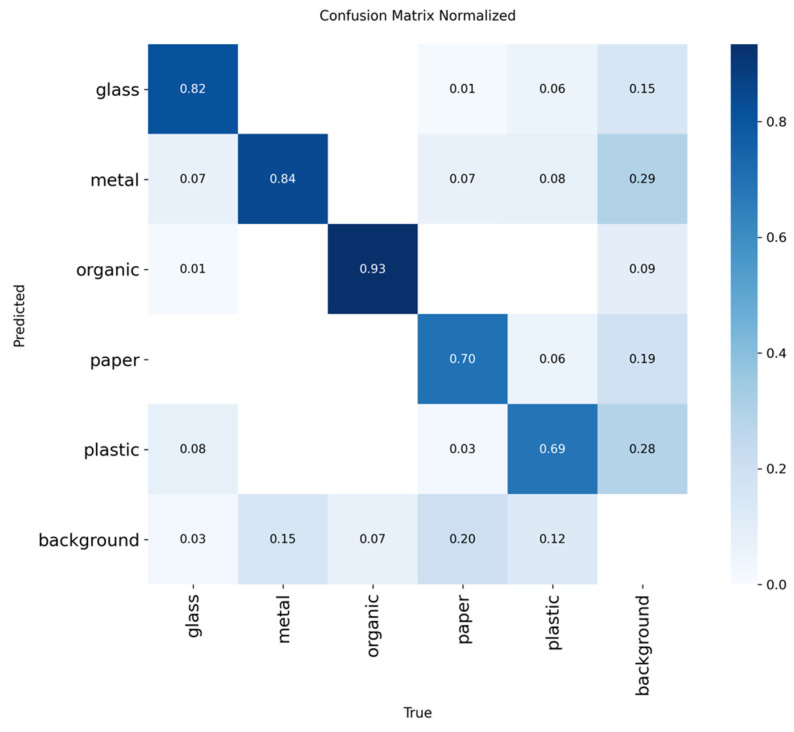
Confusion matrix generated for the YOLOv11 model.

Figure 9 shows the confusion matrix corresponding to the YOLOv11 model, which allows, in the same way as the previous one, to visualize the pattern of successes and errors in the classification of waste.

Analogous to the evaluation of YOLOv8, the performance of the YOLOv11 model on the test set was evaluated, calculating the precision, recall, F1 score, mAP@50, and mAP@50:95 metrics for each class.

This information, available in Table 10, allows us to assess in detail the model’s ability to correctly classify the different types of waste and detect possible limitations in its performance by category. The following table shows these results.

The results obtained with the YOLOv11 model show a solid and balanced performance in the classification of different types of waste.

The organic class clearly stands out, with an accuracy of 93.3% and a recall of 92.6%, which translates to an excellent F1 of 92.9% and a very high mAP@50 of 97.2%. This indicates that the model identifies this type of residue very effectively and maintains a high rate of true positives with few false positives.

The glass and metal classes also exhibit good values, with accuracy and recall above 80% and a mAP@50 that exceeds 86% for glass and 90% for metal. This reflects a robust performance in the detection and classification of these materials, which is essential for proper separation in automated recycling systems.

On the other hand, the paper and plastic classes have somewhat more modest results, with an accuracy of around 70% and a recall of around 60% along with slightly lower mAP values, especially in the mAP@50:95 range. This suggests that the model faces greater challenges in correctly identifying these materials, perhaps due to their visual variability or confusion with other classes.

Overall, the F1 score values reflect a good balance between accuracy and recall, with satisfactory performance for practical applications. The model proves to be particularly effective in detecting organic and metallic waste, which can be very beneficial for optimizing automated sorting processes in real-world environments.

#### 5.1.4. Results of the YOLOv12 Model

The YOLOv12 architecture, evaluated under the same criteria as its predecessor, showed significant improvement in several key aspects of performance. With a confidence threshold of 80%, maximum overlap of 30%, and IoU threshold of 45%, the model achieved a mAP@50 of 86.21%, which is slightly higher than that obtained by YOLOv11. This increase, although subtle, represents an improvement in the spatial accuracy of detections, which is especially relevant in an environment with small or partially overlapping objects.

**Figure 10:**
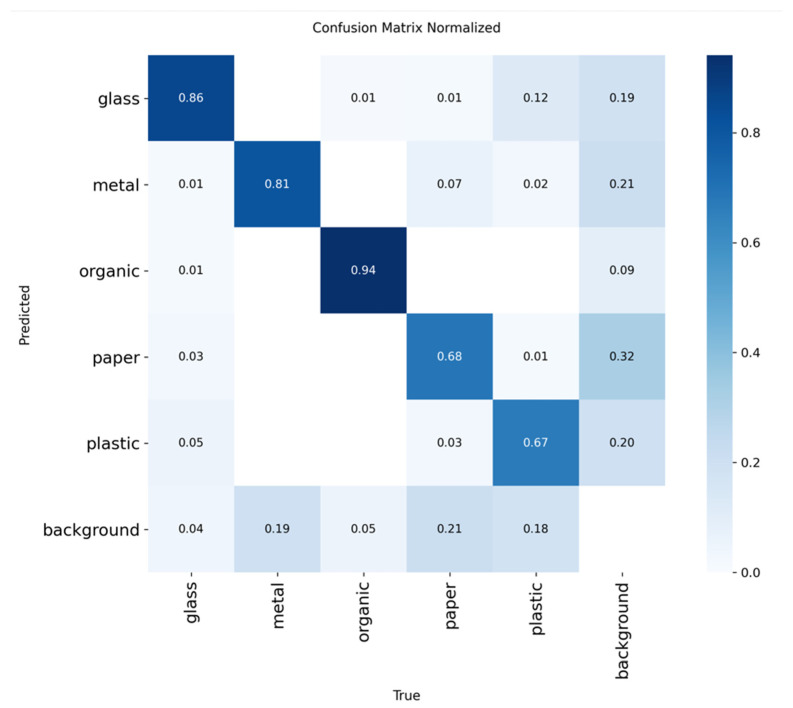
Confusion matrix generated for the YOLOv12 model.

The YOLOv12 model was evaluated on the test set, and, from the confounding matrix shown in Figure 10, key metrics (accuracy, recall, and F1 score) were calculated per class to analyze its performance in waste classification, as was carried out with the previous models, which can be seen in Table 11.

The results obtained with the YOLOv12 model, available in Table 11, show a consistent and reliable behavior in waste classification, standing out for a significant improvement in accuracy and an adequate relationship between the key evaluation metrics. The best experiment achieved an F1 score of 80.06% and a mAP@50:95 of 66.9%, which evidences an overall competitive performance, especially considering the variety of classes involved.

The organic class once again stands out as the best classified, with an accuracy of 94.0%, a recall of 93.0% and an F1 score of 93.5%, along with a very high mAP@50 of 97.8%. This indicates that the model identifies organic waste with very high reliability and minimal confusion, making it ideal for applications that prioritize this category.

Metal and glass also score robust metrics: metal achieves an accuracy of 89.9%, while glass excels in recall (90.8%), which translates to F1 scores of 83.8% and 84.8%, respectively. In addition, both classes exceed 90% in mAP@50, consolidating their reliability in automatic sorting tasks.

In contrast, the paper and plastic classes present similar challenges to those observed in YOLOv11. Paper has an F1 of 73.8%, while plastic shows a lower F1 of 67.8%, with a recall that barely exceeds 58%. These figures, together with lower mAP@50:95 values (56.1% for paper and 57.9% for plastic), suggest greater variability in their visual representation or greater ambiguity compared to other classes.

In summary, YOLOv12 offers modest but significant improvements over previous versions, especially in the robustness of the sorting of metal and organic waste. While more visually ambiguous classes remain challenging, the model proves to be an effective tool for automated recycling environments, with a good balance between accuracy and recall.

### 5.2. System Latency

Regarding performance in the inference phase, latency, i.e., the time needed for each model to process an image and generate a prediction, was evaluated during local tests with standard resolution input images (640 × 640 pixels). YOLOv8 showed a total time per image of 7.0 ms (0.3 ms preprocessing, 5.2 ms inference, and 1.5 ms post-processing), which equates to approximately 142.86 images per second (FPS). Meanwhile, YOLOv11 had a total time per image of 7.6 ms (0.3 ms preprocessing, 5.1 ms inference, and 2.2 ms post-processing), with a speed of around 131.58 FPS. Finally, YOLOv12 presented a total time per image of 8.7 ms (0.2 ms preprocessing, 7.8 ms inference, and 0.7 ms post-processing), reaching about 114.94 FPS. These results suggest that all the models evaluated are viable for near-real-time applications, such as automatic waste sorting systems on conveyor belts, recycling stations, or controlled urban environments, where speed and efficiency are key.

### 5.3. Comparison Between Versions of the YOLO Model

In order to determine which of the versions of the YOLO model is more suitable for the task of automatic sorting of municipal solid waste, a systematic comparison was made between the YOLOv8, YOLOv11, and YOLOv12 architectures. The evaluation was based on the same dataset and under homogeneous experimental conditions, keeping constant the thresholds of confidence (80%), maximum overlap (30%), and IoU (45%).

A first approximation to the comparative analysis can be obtained through the total number of true positives (TPs), false positives (FPs), and false negatives (FNs) registered by each model, broken down by class. These metrics allow you to directly assess each version’s ability to correctly identify objects (TPs), avoid misclassification (FPs), and minimize omissions (FNs). A graphical representation using bar charts could facilitate the interpretation of these results, especially with regard to the behavior by class (metal, organic, paper, plastic, and glass). However, this study chooses to focus the analysis on composite metrics that offer a more integrated view of performance.

Comparatively, the YOLOv8, YOLOv11, and YOLOv12 models demonstrated strong performances, but with notable differences in their key metrics. The YOLOv12 model stood out as the most accurate, with an accuracy of 84.60%, a recall of 75.97%, and the best overall F1 score of 80.10%, reflecting a reasonable balance between accuracy and coverage. In addition, it achieved the highest mAP@50 (86.21%) and a mAP@50:95 of 66.91%, evidencing its ability to perform consistent detections at different IoU thresholds, although with a lower processing speed (114.94 FPS), which could limit its application in very demanding real-time environments.

On the other hand, YOLOv11 presented a very competitive performance, with an accuracy of 81.79%, a recall of 78.02%, and an F1 score of 79.88%, which is just below the most recent model. Its mAP@50 reached 83.32% and its inference speed was slightly higher (131.58 FPS), making it a balanced choice for tasks where good coverage is required without sacrificing too much operational performance.

Finally, the YOLOv8 model, although with the most modest metrics among the three, maintained an accuracy of 77.75%, a recall of 79.63%, and an F1 score of 78.68%, reaching a mAP@50 of 82.87% and a mAP@50:95 of 64.40%. Its main advantage was performance in speed, achieving 142.86 FPS, which makes it especially suitable for systems with limited computational resources or applications that require near-real-time responses.

For a more detailed comparison, the consolidated values, in Table 12, of the key metrics for each version of the model are presented below:

The F1 score, being the harmonic mean between accuracy and recall, provides a balanced measure between the model’s ability to correctly detect recall and its accuracy in doing so (accuracy). In the current analysis, although YOLOv12 presents the highest accuracy (84.60%) and the best performance in mAP@50 (86.21%) and mAP@50:95 (66.91%), it is the YOLOv11 model that achieves the highest F1 score (80.10%), which shows a better overall balance between detection and correct classification.

In short, the choice between the different versions of the model will depend on the operational context in which they are implemented. YOLOv12 is ideal for systems where the priority is to minimize sorting errors, such as automated industrial applications with minimal human supervision. On the other hand, YOLOv11 could be preferred in scenarios where a more complete detection of residues is valued, even if this means a small increase in false positives. Finally, YOLOv8 remains a solid choice for environments with hardware constraints or high-speed processing needs, thanks to its excellent FPS performance (142.86), albeit with a slight reduction in its accuracy and coverage metrics.

Given the objective of this study, YOLOv12 was chosen as the final model, as it provides the best overall results in terms of precision and accuracy, and the slight increase in response time does not represent a significant limitation in the context considered. However, in other scenarios with tighter real-time constraints or limited computational resources, older versions such as YOLOv11 or YOLOv8 could be considered more suitable for those specific requirements. Based on the overall results presented, we now proceed to analyze the specific behavior of each model according to the different types of waste. To this end, three bar graphs have been developed illustrating the accuracy, recall, and F1 score metrics obtained for each metal, organic, paper, plastic, and glass class in the YOLOv8, YOLOv11, and YOLOv12 versions. These visualizations allow you to more clearly identify the relative strengths and weaknesses of each architecture in relation to specific types of waste.

**Figure 11:**
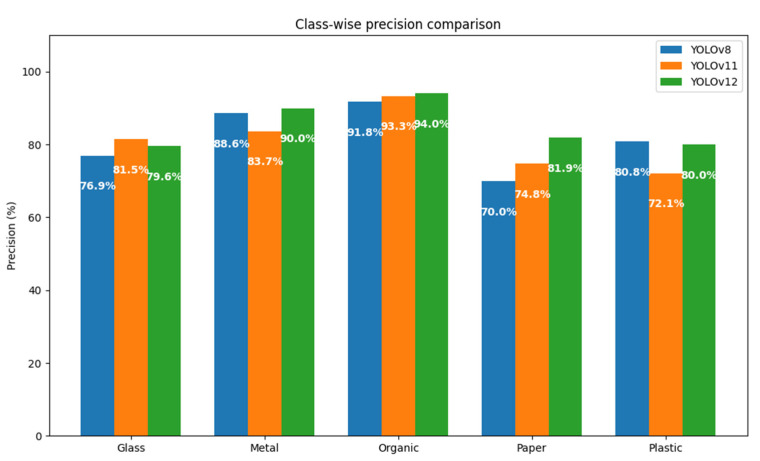
Precision metric comparison.

The graph in Figure 11 shows a comparison of accuracy by class between three versions of the YOLO model: YOLOv8, YOLOv11, and YOLOv12. The comparison was applied to the classification of five types of waste: glass, metal, organic, paper, and plastic. Accuracy, expressed as a percentage, indicates how effectively each model correctly identifies objects in each class.

In general terms, YOLOv12 presents the best performance, standing out in the metal (90.0%), organic (94.0%), and paper (81.9%) classes. YOLOv11 slightly outperforms the other models in the glass class (81.5%), while YOLOv8 scores its best result in plastic (80.8%), narrowly beating YOLOv12. Although YOLOv11 shows improvements over YOLOv8 in some classes, its results are less consistent than those of YOLOv12.

The evolution of the versions shows progress in the ability to detect and classify, especially in more challenging classes such as paper, where YOLOv12 is significantly improved over previous versions. In summary, the graph shows that YOLOv12 offers the highest overall accuracy, being a more robust and reliable option for automated waste sorting applications.

**Figure 12:**
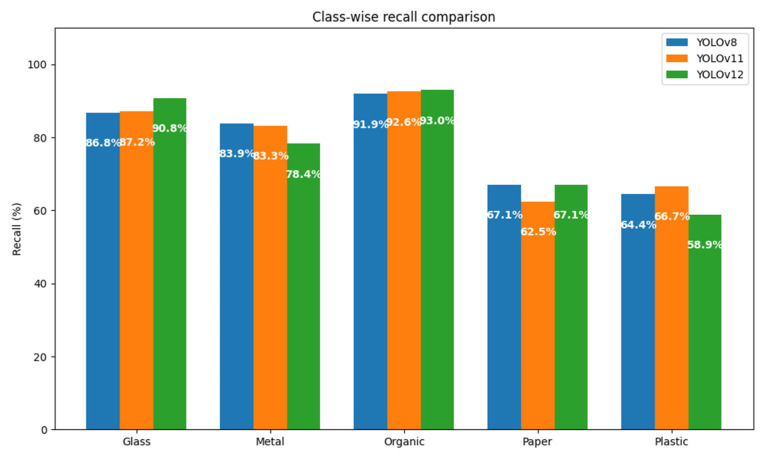
Recall metric comparison.

Figure 12 shows a class recall comparison between three versions of the YOLO model: YOLOv8, YOLOv11, and YOLOv12. The comparison was applied to the classification of five types of waste: glass, metal, organic, paper, and plastic. The recall, expressed as a percentage, indicates the model’s ability to correctly retrieve the elements of each class, i.e., how well it identifies all relevant objects without omitting any.

Overall, YOLOv12 presents the strongest performance, standing out in the glass (90.8%), organic (93.0%), and paper (67.1%) classes, equaling YOLOv8 in the latter. YOLOv11 slightly outperforms the other versions only in the plastic class (66.7%), while YOLOv8 obtains its best result in metal (83.9%), where YOLOv12 shows a noticeable drop (78.4%).

Although YOLOv11 improves over YOLOv8 in some classes, its results are less consistent. The evolution towards YOLOv12 reflects an advance in the more accurate detection of residues, with particular improvement in complex classes such as glass and organic. Overall, the results show that YOLOv12 offers better overall recall, making it a more reliable choice for automated waste sorting tasks.

**Figure 13:**
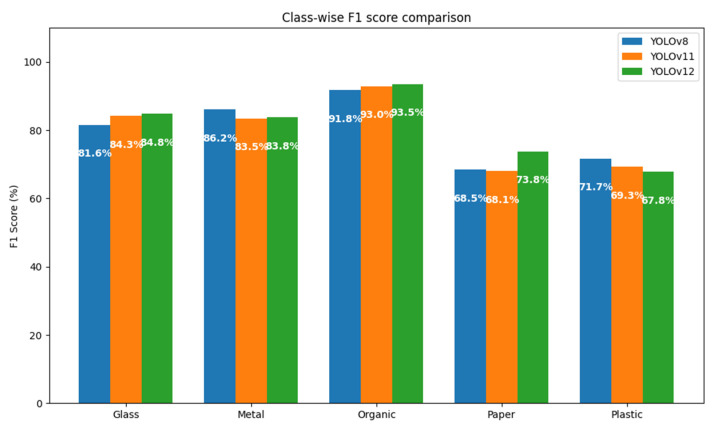
F1 score metric comparison.

The other graph shown in Figure 13 shows a comparison of the F1 score by class between the YOLOv8, YOLOv11, and YOLOv12 models. This comparison was applied to the classification of waste into five categories: glass, metal, organic, paper, and plastic. The F1 score, expressed as a percentage, combines accuracy and recall into a single metric, offering a balanced assessment of the model’s performance in each class.

Overall, YOLOv12 presents the most consistent and elevated performance, leading in the organic (93.5%) and paper (73.8%) classes. In the glass category, YOLOv11 obtains a slightly higher result (84.3%) compared to YOLOv12 (84.0%) and YOLOv8 (81.6%). For metal, the results are fairly even, with YOLOv8 leading the way with 86.2%, closely followed by YOLOv12 (83.8%) and YOLOv11 (83.5%).

In the plastic class, although the differences are smaller, YOLOv8 achieves the highest F1 score (71.7%), slightly surpassing YOLOv11 (69.3%) and YOLOv12 (67.8%). Despite these ups and downs, YOLOv12 shows the best overall average, suggesting greater robustness in different types of waste. This evolution indicates significant progress towards more accurate and balanced models in automated classification tasks.

### 5.4. Test Case of the Verification System

To conclude the results section, a real use case of the verification system is presented, with the aim of illustrating the practical application of the trained models in the functional environment of the developed web platform. This interface allows the user to start the recycling process and visualize the result of the automatic sorting in real time.

**Figure 14:**
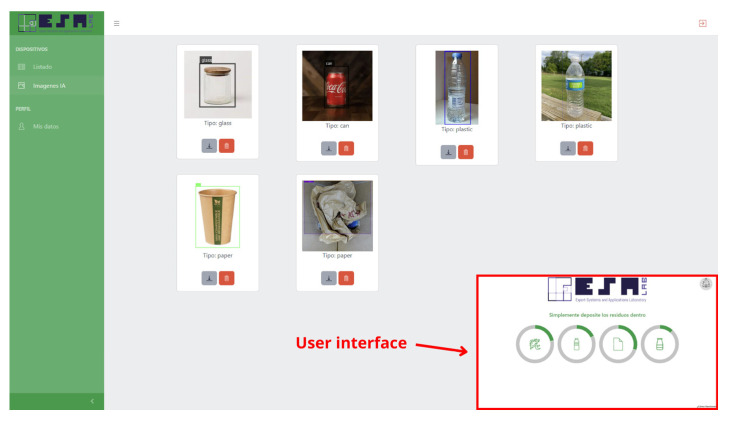
System web interface showing sorted waste and the filling status of containers using circular indicators.

Figure 14 shows a screenshot of the sorting interface, where the detected waste is identified and labeled according to its corresponding class. Each object is categorized based on the predictions made by the selected YOLO model, allowing the user to validate the classification before proceeding with the disposal of the waste. In addition, a circular indicator represents the current fill level of each of the containers (glass, plastic, metal, organic, and paper), providing a clear and intuitive display of the occupancy status of each tank.

**Figure 15:**
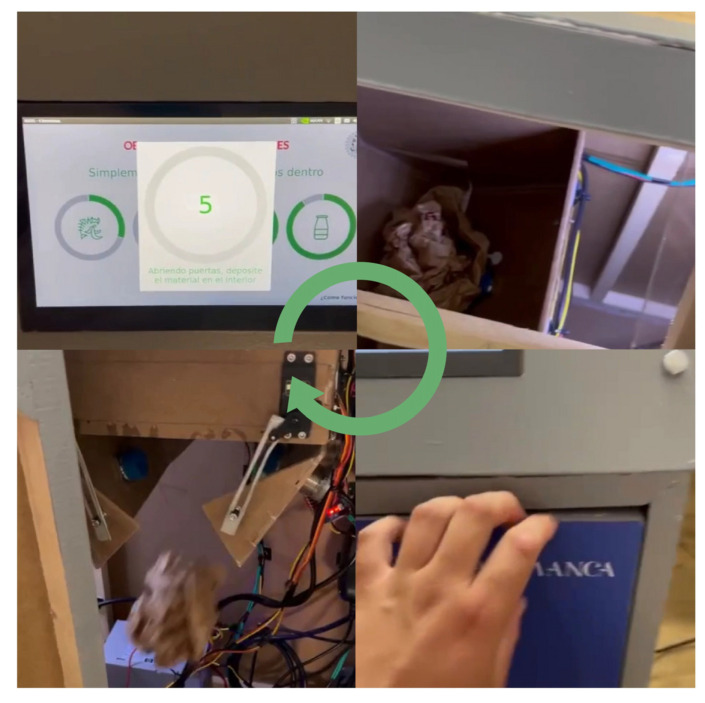
Image of the recycling process, where waste is sorted and automatically redirected to its corresponding container.

Figure 15 shows a second screenshot illustrating the recycling process in action. Here, a recognized piece of waste is correctly sorted and assigned to its corresponding container, which validates the complete cycle of detection, classification, and physical assignment of the waste.

This case study highlights the effectiveness of the system in a real-world environment, where user interaction, model accuracy, and system responsiveness converge, thus contributing to an automated and intelligent waste management process. In addition, as previously mentioned in the architecture proposal, for a better understanding of the process, a video of the operation can be seen at the link provided in [50].

## 6. Discussion

In this section, the results obtained are analyzed and interpreted, and the broader context of the data is studied. Aspects related to daily waste statistics and their social impact are included, which complement the experimental evaluation presented previously. This analysis allows for a better understanding of the relevance and practical implications of the proposed model.

**Figure 16:**
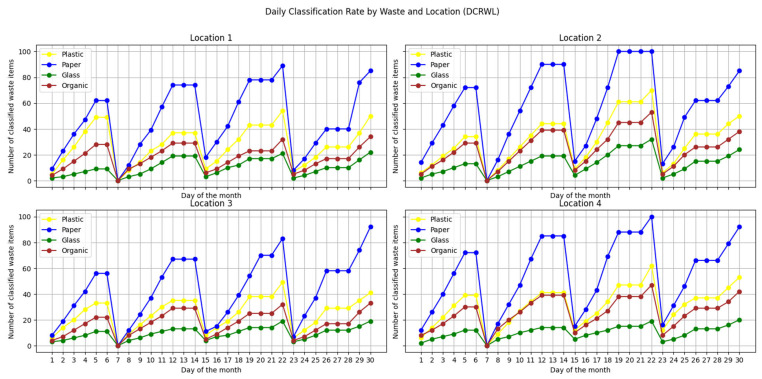
Daily evolution of waste classification by type in each location.

**Figure 17:**
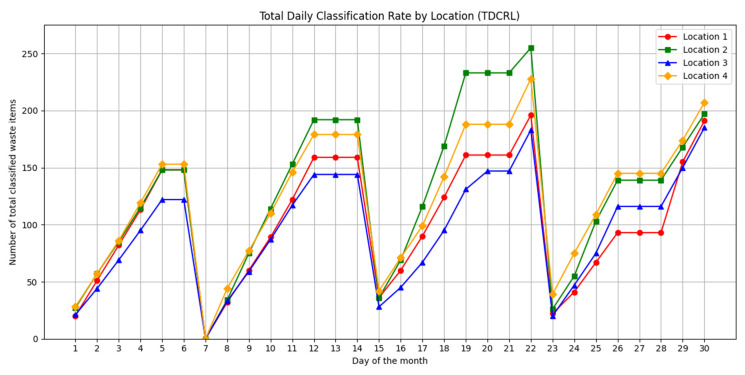
Total daily waste sorting rate by location.

**Figure 18:**
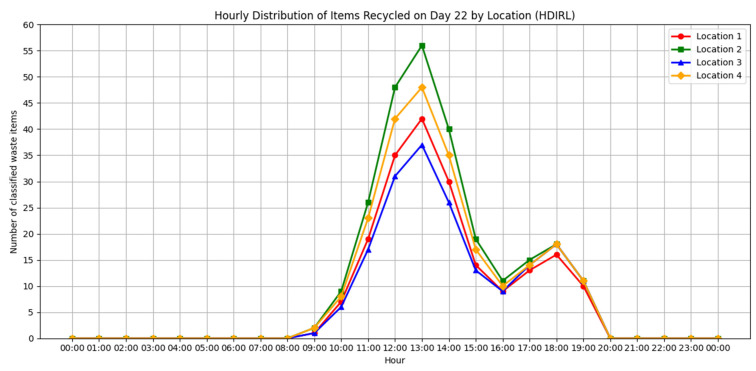
Average hourly frequency of recycling by location on the busiest day.

This section analyses three key metrics that allow us to assess waste sorting behavior in different locations. The “Daily Sorting Rate by Waste at Each Location (DCRWL)” shows the daily distribution of sorted waste, disaggregated by type and disposal point, allowing the variability in sorting according to the waste category to be observed (see Figure 16). The “Total Daily Sorting Rate by Location (TDCRL)” reflects the temporal evolution of the total waste sorted at each location, making it easier to identify general trends (see Figure 17). Finally, the “Average Hourly Frequency by Location (HDIRL)” represents the average usage behavior per time slot in each location, allowing the detection of the times of the day with the greatest activity in the classification (see Figure 18).

### 6.1. Results of Data Collection

In this section of results, the results obtained from the metrics previously established in the previous section of the case study are described. Through these four previously defined rates, we have been able to observe in the different graphs that the recycling rate has been increasing over the days that the prototype has been present.

It is also important to note that the sorting prototype is not very large; we emptied the prototype approximately every seven days or eight days.

It should be added that this study was carried out in four public workspaces with a schedule of activity from Monday to Friday, from 8:00 a.m. to 8:00 p.m. Therefore, during the weekends, the recycler was not used, and the emptying was only carried out if the scheduled date coincided with a Saturday or Sunday.

It is important to bear in mind that recyclers have a maximum average capacity of 100 items per container, which means that, once this threshold is reached, the waste deposit is interrupted until the corresponding emptying is carried out. In order to guarantee the correct functioning of the system, the recyclers are emptied periodically during the night on the following dates: from the 6th to the 7th, from the 14th to the 15th, from the 22nd to the 23rd, and from the 30th to the 1st of the following month.

#### 6.1.1. Daily Sorting Rate per Waste at Each Location

This metric represents the daily behavior of the automatic waste sorting process in four different locations, where the smart recycler has been installed. That is, each location where the recycler has been installed is shown as “Location 1”, “Location 2”, “Location 3”, or “Location 4”.

In Figure 16, each subgraph shows one for each location, showing the daily evolution of the volume of waste classified. For each type of waste, the number of items recycled per day over a 30-day month is represented. This information makes it possible to visualize the frequency with which users deposit each type of waste in the machines and allows the identification of usage patterns and recycling preferences. Flush times result in visible restarts in the data, as the waste count is reset with each flush operation.

From the analysis of the graphs and the experimentation carried out, the following general conclusions can be drawn:

- Paper and plastic are the most frequently recycled waste. In all locations, these two materials have the highest daily classification values, which suggests a greater availability of this waste by users or a greater ease of separation.
- Glass is the least commonly deposited waste. Its values are consistently low in all locations, which could be attributed to a lower generation of this type of waste in the environment or to the lower willingness of users to transport it to the recyclers.
- The behavior by location is relatively constant, although there are slight variations in the total daily volumes, which are probably related to geographical location, the influx of people, or the recycling habits of the population.
- Cyclical patterns are observed in the accumulation of waste, which is clearly influenced by the empty moments. After each emptying operation, the sorted quantities begin to increase again, following a trend very similar to previous cycles. This not only reflects the regularity in the use of the recyclers, but also the efficiency of the maintenance and emptying system implemented.

Daily graphs by waste type allow you to clearly visualize usage trends and accumulation dynamics at each location, confirming repetitive patterns linked to landfills and making it easier to identify opportunities to optimize recycling management.

#### 6.1.2. Daily Total Classification Rate by Location

Figure 17 shows the evolution of the total daily waste sorting rate in the four locations. In this case, the total sum of items classified per day is represented, considering together the four types of waste processed: plastic, paper, glass, and organic.

Each line corresponds to a location and allows the total volume of waste managed daily in each recycling plant to be visualized in an aggregated way. This overview makes it easy to compare the overall activity at each collection point over the course of a month, as well as to detect potential fluctuations or changes in user behavior.

Based on the analysis of the graph, the following relevant observations stand out:

- Total accumulation curves reflect peaks and dips that coincide with machine emptying times, demonstrating that the maintenance system is operating regularly and on schedule.
- Although the behavior is similar between locations, variations can be seen in the total daily volumes, which suggests differences in the level of citizen participation, in the flow of people, or in the generation of waste, depending on the environment of each recycler.
- The global representation allows the identification of filling cycles, where the volume of waste increases progressively until a threshold is reached, followed by an abrupt drop related to emptying. This dynamic is repeated throughout the month, giving rise to a periodic structure in the data.

This graph serves as a complement to the analysis by type of waste presented in Section 6.1.1, as it offers a global view of the daily use of each recycling point. In general, a similar trend is observed in all locations, with a progressive accumulation of waste that restarts after the scheduled emptying. This dynamic of growth and decline coincides with what has already been seen in the graphs by type of waste.

#### 6.1.3. Average Hourly Frequency

Figure 18 shows the hourly distribution of the recycled items over a specific day, which was selected because it is the day on which the highest total amount of recycled waste was recorded among all the locations (in this case, the 22nd of the month).

The analysis of the graph allows us to observe the times of the day when users deposit the greatest amount of waste in the smart recycler, identifying two main peaks in daily activity. These peaks coincide with the hours close to noon and evening that correspond to the times when more people use the machines, which is probably related to work breaks or transfers between activities.

During the hours outside working hours (before 8:00 a.m. and after 8:00 p.m.), recycling activity is null, which coincides with the hours of operation established for the spaces where the recyclers were installed. This confirms that the use of machines is closely linked to the presence and movement of people during the working day.

In addition, the hourly frequency curve presents periods with low or no activity, which can be interpreted as breaks or times when users do not interact with the recyclers. The double-peak shape reflected in the graph shows a pattern of use that can be used to optimize the management and maintenance of the machines, as well as to plan awareness campaigns aimed at increasing participation.

In summary, this graph offers a detailed view of the daily recycling behavior in each location, showing how the interaction of users with the recyclers follows consistent temporal patterns related to work schedules and human activity.

#### 6.1.4. Error Rate

One of the fundamental aspects of assessing the reliability and accuracy of the automatic waste sorting system is the error rate analysis. This metric allows us to identify the proportion of items that have been incorrectly classified by the system compared to a manual review carried out after each emptying of the recyclers.

During the study, a post-discharge verification protocol was established by the recycler. In each programmed emptying, carried out approximately every seven or eight days, all the waste classified by the system was manually inspected in order to contrast the results obtained by artificial intelligence (AI) with the real categorization of the deposited objects.

This manual validation process consisted of individually reviewing the items contained in each compartment of the recycler to detect possible classification errors, such as the presence of non-corresponding elements (for example, organic remains erroneously deposited in the plastic compartment or paper located in the glass container).

The main purpose of this methodology was to evaluate the effective success rate of the system under real conditions of use and compare it with the accuracy rate reported by the AI model used for classification. In theory, both rates should coincide and have very similar values, since the AI is in charge of making the final decision on the category of each waste. However, factors such as ambient lighting, the orientation of the waste at the time of image capture, or external interference can influence the system’s decision-making, generating occasional discrepancies.

Table 5 shows the results obtained in the automatic detection and classification of recyclable materials, during day 22 of each study, compared to the manual counting carried out in the emptying of the recycler. Data are presented for four different locations and four types of materials: plastic, paper, glass, and organic.

The success rate reflects the accuracy of the model in correctly identifying each material compared to manual counting, identifying the difference in materials. The results show a consistent and high performance in all locations, with success percentages ranging from 79.5% to 95.2%.

Particularly noteworthy are the high accuracy rates in the classification of glass and plastic, while paper presents a little more variability, being the material with the lowest percentage in some locations. This analysis, which can be seen in Table 13, confirms the effectiveness of the model to support automated processes in the management and classification of recyclable waste.

In terms of overall performance by location, the average success rate remains consistently high in all the locations evaluated. “Location 1” has an average success of 87.8%, “Location 2” reaches 88.9%, “Location 3” 84.5%, and “Location 4” shows 85.9% average accuracy.

These values reflect that the model maintains a robust performance in different conditions and locations, confirming its ability to correctly classify most of the recyclable materials detected. However, although the percentage of success is high, additional adjustments and improvements are required to try to achieve accuracy close to 100%, since in recycling processes it is essential to maximize the recovery and correct classification of all materials and thus guarantee the sustainability of the system.

## 7. Limitations of the Proposed Approach

Despite the positive results obtained with the developed smart bin prototype, it is important to point out some limitations of the current approach that will need to be addressed in future versions of the system.

Firstly, from a technical point of view, the performance of the system is conditioned by the capabilities of the hardware used for both training and commissioning the device. The use of the Jetson Nano microcomputer, although it represents an efficient and economical solution, imposes certain restrictions in terms of simultaneous processing of tasks, especially if you want to expand the number of classes detected or apply more complex deep learning models.

In addition, the accuracy of the machine vision system can be affected by extreme lighting conditions or deformed, dirty, or partially hidden debris, limiting its robustness in highly variable environments. To try to solve the lighting problem, an LED strip was finally incorporated to improve the illumination of the images captured by the camera.

Moreover, although the overall classification results are satisfactory, variable accuracy was observed by class. In particular, categories such as paper or glass presented lower F1 scores than other classes, suggesting that certain types of residues represent specific challenges to the model. These differences may be due to variations in the shape, transparency, or reflectance of the materials, as well as their lower relative presence in the dataset.

Another important limitation lies in the size and diversity of the dataset used to train the models. Although a dataset of its own was built with real images, it can still be considered limited compared to large-scale public databases. This can influence the generalization of the model when faced with scenarios not contemplated during the training phase.

From an operational point of view, the evaluation of the system was carried out in public workspaces, such as universities, over a period of four months, which, although it allowed its functionality to be validated, does not guarantee its behavior in environments with a much higher density of use, such as public transport stations or festivals, or under adverse weather conditions or vandalism.

As for the implemented private blockchain, although the use of the Proof of Authority (PoA) algorithm significantly reduces energy consumption and improves performance, the system depends on an active and maintained network infrastructure, which can make it difficult to replicate the system in environments with limited connectivity or where technological maintenance costs are a barrier.

Finally, it is worth highlighting the difficulty inherent in the development of a system of these characteristics without starting from a previous design or direct reference. The prototype was conceived and built entirely from scratch, hand-designing both the mechanical drawings and the internal separation mechanisms, electronic integration, and housing. This approach, while giving flexibility and creativity to the design, also involved considerable additional effort in time, iterations, and functional validation, compared to proposals that start from existing systems.

These limitations do not invalidate the results obtained, but they do mark a clear path for improvement to strengthen the proposal and guarantee its applicability in real, large-scale scenarios.

## 8. Conclusions and Future Lines

This work presents an innovative technological solution for the intelligent management of urban solid waste through the development of an automatic sorting system based on computer vision, physical sensors, multi-agent systems, and blockchain technology. The system was conceived under the principles of low cost, modularity, and replicability, with the aim of facilitating its adoption in real contexts such as educational, domestic, or institutional environments.

The distributed architecture, implemented on the PANGEA platform, allows each functional component of the system—sensors, actuators, camera, decision logic, and registration—to operate as an autonomous intelligent agent. This structure favors scalability, decentralized maintenance, and the adaptation of the system to different operational scenarios. In parallel, the integration of an Ethereum-based private blockchain network ensures the traceability, immutability, and auditability of classification events using a Proof of Authority (PoA) consensus mechanism.

The physical prototype, built entirely from low-cost hardware and widely available components, including a Jetson Nano microcomputer, inductive and capacitive sensors, NEMA 17 motors, and a Logitech Brio 4K camera, reached a total cost of less than EUR 1000, supporting its economic viability and potential for large-scale replicability.

In quantitative terms, the computer vision model implemented in the final prototype, YOLOv12, achieved an overall accuracy of 84.6%, a recall of 81.0%, and a mAP@50 of 86.2%, demonstrating a high ability to correctly identify the different types of waste. System latency during the testing phase was maintained below 9 ms per image, ensuring a near-real-time response suitable for use in everyday environments and utilitarian embedded systems. During the four months of evaluation, a progressive increase of up to 20% in the correct classification rate of recyclable waste was observed, indicating a positive impact on user behavior. The total cost of the prototype remained below EUR 1000, which supports the economic viability of the system for real applications.

From the point of view of sustainability, the experimental results show that this system has a direct and positive impact on several Sustainable Development Goals (SDGs). Specifically, the identified goals are as follows:

- SDG 9 (Industry, Innovation and Infrastructure): The technical architecture based on a MAS and blockchain demonstrates that it is possible to design and deploy an intelligent, distributed, and resilient infrastructure with limited resources. The low cost of the prototype (EUR 974), its modularity, and the integration of advanced technologies support responsible and accessible innovation, aligned with sustainable infrastructures.
- SDG 11 (Sustainable Cities and Communities): The implementation of the system in four different urban locations over four months allowed us to observe a pattern of regular use and an increase in the recycling rate. This behavior reflects an improvement in waste management at the local level and greater efficiency in separation at source, reducing the pressure on municipal collection and treatment infrastructures.
- SDG 12 (Responsible Consumption and Production): Automatic sorting, with an accuracy of more than 85% and a progressive improvement of 20% in the correct separation rate, helps to prevent recyclables from ending up in landfills. The system promotes more efficient management of resources, reinforcing the principles of the circular economy.
- SDG 13 (Climate Action): Automating the separation process and improving the recovery of materials contributes to reducing untreated waste and emissions from final disposal. Traceability and data analysis also make it possible to design evidence-based management policies to mitigate environmental and climate impacts.

As future lines of research, the optimization of the adaptive behavior of the multi-agent system through the use of reinforcement learning techniques is proposed, allowing agents to adjust their decisions based on dynamic operating patterns. Likewise, the integration of advanced sensors, such as spectrometers, is contemplated to address the classification of complex or contaminated waste.

Another strategic direction is the interconnection of multiple units through a common IoT infrastructure, in order to generate dynamic waste generation maps and enable predictive collection strategies. Finally, it is proposed to explore tokenized incentive systems through smart contracts, which reward citizen participation and promote sustainable practices through gamification mechanisms and the digital circular economy.

Finally, we can say that the developed system constitutes a relevant and applicable technological contribution, which combines technical effectiveness, economic efficiency, and alignment with global sustainability objectives. Its implementation in real scenarios can play a transformative role in urban waste management, facilitating transitions towards smarter, cleaner, and more sustainable cities.

## Figures and Tables

**Table 1:** Key feature comparison between the PANGEA and JADE multi-agent systems.

Features	Pangea	JADE
Support for organizations	Native and explicit	Limited, requires manual extensions
RFC-based communication	Similar style to web protocols	No, use FIFA-ACL (more closed)
Focus on virtual organizations	UO-oriented design	Not directly focused on individual agents
Organizational scalability	High (with hierarchies and roles)	Stocking
External interoperability	High (by use of network standards)	Low (requires adaptations)
Modularity	High (decoupled components)	Stocking
Community and documentation	More academic and specialized	Larger and industrially supported

**Table 2:** List of materials for the construction of the prototype.

Category	Component/Material	Description	Estimated Price (EUR)
Processing Unit	Jetson Nano (NVIDIA Corporation, Santa Clara, CA, USA)	AI microcomputer for image/model processing	EUR 300
	ESP8266 (Espressif Systems, Shanghai, China)	Wi-Fi microcontroller for sensor data	EUR 10
Sensing and Vision	Logitech Brio (Logitech Inc., Newark, CA, USA)	4K UHD webcam with HDR and autofocus	EUR 215
	Specialized physical sensors (sourced from BricoGeek, Ponferrada, León, Spain)	Inductive and capacitive	EUR 70
Actuators and Motion	NEMA 17 Stepper Motor (sourced from BricoGeek, Ponferrada, León, Spain)	High-precision motion control	EUR 30
	DC motors (x2) (sourced from BricoGeek, Ponferrada, León, Spain)	Motorized gate and tray movement	EUR 18
	L298N motor driver (STMicroelectronics, Geneva, Switzerland—generic module)	Dual H-bridge motor controller	EUR 6
	Timing belt and pulleys	Mechanical transmission for the tray	EUR 25
User Interface	Touchscreen display	5–7″ touchscreen for system interaction	EUR 100
Power and Wiring	Power supply unit	Supplies power to the system	EUR 50
	Cables (signals, power, and data)	Wires for sensors, motors, display, and camera	EUR 30
Construction Materials	MDF/plywood	Chassis and housing materials	EUR 80
	Screws, bolts, and fasteners	For structural assembly	EUR 15
	Rails, brackets, and guides	Structural components for movement and support	EUR 25
Total			EUR 974.00

**Table 3:** Distribution of annotations by class.

Class	Annotations	Train	Valid	Test
Metal	2816	2129	222	465
Organic	770	499	136	135
Glass	326	226	76	24
Paper	591	479	76	36
Plastic	369	248	90	31
Total	4872	3581	600	691

**Table 4:** Fields presented in each blockchain record.

Field	Description
Id	A unique identifier generated by a hashed algorithm (SHA-256) used to avoid collisions and ensure the uniqueness of the event.
numSeries	The serial number of the capture device, used for traceability of the hardware involved in collection.
localization	The geographical coordinates (latitude and longitude in decimal format) of the exact point of collecting the waste.
typeWaste	The coded string represents the category of waste identified by the machine vision system.
quantity	The number of individual elements or homogeneous units detected in the classification event.
DateTime	A timestamp in UNIX format that indicates the exact moment of the event, ensuring cross-platform compatibility.
active	A boolean value that represents the operational state of the system at the time of the event (active = true/inactive = false).

**Table 5:** Number of images in the dataset.

Total Images	Train (75%)	Validation (11%)	Test (14%)
3134	2362	334	438

**Table 6:** Hardware and software specifications.

Category	Specification
Operating System	Windows 11 (64-bit)
Graphics Processing Unit (GPU)	NVIDIA GeForce RTX 4050 (6 GB dedicated)
Processor (CPU)	Intel Core i7-13620H (13th gen, 10 cores/16 threads) (Intel, Santa Clara, CA, USA)
RAM Memory	16 GB DDR4
Execution Environment	Windows 11
Programming Language	Python 3.13.5
Libraries Used	PyTorch 2.7.1, NumPy 2.3.0

**Table 7:** Better hyperparameters of the grid search for each model.

Experiment	Times	Batch Size	Learning Rate
YOLOv8	50	8	0.001
YOLOv11	50	8	0.01
YOLOv12	50	16	0.01

**Table 8:** Constant training parameters during grid search.

Parameter	Value
Image size	640 × 640
Momentum	0.937
Weight decay	0.0005
Scale	0.5
Mosaic	1.0
FlipLR	0.5

**Table 9:** Results by class for the YOLOv8 model.

Class	Accuracy (%)	Recall (%)	F1 (%)	mAP@50 (%)	mAP@50:95 (%)
Glass	76.93	86.84	81.58	90.16	66.91
Metal	88.58	83.86	86.16	91.78	78.02
Organic	91.75	91.91	91.83	96.50	79.55
Paper	69.97	67.11	68.51	72.83	47.13
Plastic	80.82	64.44	71.71	73.60	53.95

**Table 10:** The results by class for the YOLOv11 model.

Class	Accuracy (%)	Recall (%)	F1 (%)	mAP@50 (%)	mAP@50:95 (%)
Glass	81.54	87.19	84.27	86.40	63.25
Metal	83.70	83.29	83.50	90.51	77.74
Organic	93.33	92.58	92.95	97.21	82.01
Paper	74.80	62.51	68.10	76.89	51.93
Plastic	72.12	66.67	69.29	76.49	55.47

**Table 11:** The results by class for the YOLOv12 model.

Class	Accuracy (%)	Recall (%)	F1 (%)	mAP@50 (%)	mAP@50:95 (%)
Glass	79.58	90.79	84.82	90.87	64.41
Metal	89.96	78.38	83.77	90.85	79.43
Organic	94.05	93.00	93.52	97.77	80.74
Paper	81.94	67.11	73.78	79.77	56.12
Plastic	79.98	58.89	67.83	78.57	57.90

**Table 12:** Global metrics of trained YOLO versions.

Model	Accuracy (%)	Recall (%)	F1 Score (%)	mAP@50 (%)	mAP@50:95 (%)	FPS
YOLOv8	77.75	79.63	78.68	82.87	64.40	142.86
YOLOv11	81.79	78.02	79.88	83.32	64.99	131.58
YOLOv12	84.60	75.97	80.10	86.21	66.91	114.94

**Table 13:** Analysis of error and performance of the model in material classification.

Location	Material	Material Count (Auto)	Material Count (Manual)	% Success (Manual)
Location 1	Plastic	54	48	88.9%
	Paper	89	71	79.8%
	Glass	21	20	95.2%
	Organic	32	28	87.5%
Location 2	Plastic	70	62	88.6%
	Paper	100	81	81.0%
	Glass	32	28	87.5%
	Organic	53	47	88.7%
Location 3	Plastic	49	44	89.8%
	Paper	83	66	79.5%
	Glass	19	16	84.2%
	Organic	32	27	84.4%
Location 4	Plastic	62	56	90.3%
	Paper	100	82	82.0%
	Glass	19	16	84.2%
	Organic	47	41	87.2%

## Data Availability

Publicly available datasets were analyzed in this study. This data can be found here: On a GitHub repository (https://github.com/AnOrdinaryUsser/trash5, accessed on 8 July 2025).
